# Satellite Tagging and Biopsy Sampling of Killer Whales at Subantarctic Marion Island: Effectiveness, Immediate Reactions and Long-Term Responses

**DOI:** 10.1371/journal.pone.0111835

**Published:** 2014-11-06

**Authors:** Ryan R. Reisinger, W. Chris Oosthuizen, Guillaume Péron, Dawn Cory Toussaint, Russel D. Andrews, P. J. Nico de Bruyn

**Affiliations:** 1 Mammal Research Institute, Department of Zoology and Entomology, University of Pretoria, Pretoria, South Africa; 2 Centre for Statistics in Ecology, Environment and Conservation, Department of Statistical Sciences, University of Cape Town, Cape Town, South Africa; 3 School of Fisheries and Ocean Sciences, University of Alaska Fairbanks, Fairbanks, Alaska, United States of America; 4 Alaska SeaLife Center, Seward, Alaska, United States of America; Musee National d'Histoire Naturelle, France

## Abstract

Remote tissue biopsy sampling and satellite tagging are becoming widely used in large marine vertebrate studies because they allow the collection of a diverse suite of otherwise difficult-to-obtain data which are critical in understanding the ecology of these species and to their conservation and management. Researchers must carefully consider their methods not only from an animal welfare perspective, but also to ensure the scientific rigour and validity of their results. We report methods for shore-based, remote biopsy sampling and satellite tagging of killer whales *Orcinus orca* at Subantarctic Marion Island. The performance of these methods is critically assessed using 1) the attachment duration of low-impact minimally percutaneous satellite tags; 2) the immediate behavioural reactions of animals to biopsy sampling and satellite tagging; 3) the effect of researcher experience on biopsy sampling and satellite tagging; and 4) the mid- (1 month) and long- (24 month) term behavioural consequences. To study mid- and long-term behavioural changes we used multievent capture-recapture models that accommodate imperfect detection and individual heterogeneity. We made 72 biopsy sampling attempts (resulting in 32 tissue samples) and 37 satellite tagging attempts (deploying 19 tags). Biopsy sampling success rates were low (43%), but tagging rates were high with improved tag designs (86%). The improved tags remained attached for 26±14 days (mean ± SD). Individuals most often showed no reaction when attempts missed (66%) and a slight reaction–defined as a slight flinch, slight shake, short acceleration, or immediate dive–when hit (54%). Severe immediate reactions were never observed. Hit or miss and age-sex class were important predictors of the reaction, but the method (tag or biopsy) was unimportant. Multievent trap-dependence modelling revealed considerable variation in individual sighting patterns; however, there were no significant mid- or long-term changes following biopsy sampling or tagging.

## Introduction

Cetaceans spend the vast majority of their lives under water and are highly mobile and often wide-ranging, which makes them a challenging taxon to study. Two field methods – tissue biopsy sampling and satellite-linked telemetry (or satellite tagging) – are becoming widely used in cetacean studies because they allow the collection of data which are difficult or impossible to obtain by other means. Tissues obtained by biopsy sampling can be used for a range of analyses including genetics, stable isotopes, fatty acids, contaminants, hormones and trace elements (see [1] for a review) and can so address aspects such as population structure, diet and animal health (e.g., [2]–[5]). Satellite tagging can elucidate the movement, distribution, behaviour and habitat use of cetaceans in relation to their physical environment (e.g., [6]–[8]). Such data are critical to understanding the ecology of a species and its environmental role and, consequently, are vital to conservation or management efforts (e.g., [9], [10]). The need for such information is particularly acute given the anthropogenic pressures many such populations and species face [10]–[12].

However, researchers must carefully consider their methods not only from an animal welfare perspective, but also to ensure the scientific rigour and validity of their results. The latter point is critical where methods may affect the subsequent behaviour or performance of individuals, thereby biasing the results obtained (e.g., [13]–[15]). From an ethical perspective researchers have an onus to assess the tradeoffs between the ‘importance’ of research, its likely benefit and its effect on animals before conducting work [16], [17]; from a scientific perspective the responsibility is to design robust and valid studies [18]. Researchers should further evaluate animal effects and research methods *post-hoc*, refine these where needed and, importantly, publish such results [19], [20].

Small cetaceans may be captured and restrained for satellite tagging and biopsy sampling (e.g., [21], [22]) but this is impractical for most species and therefore remote techniques, which employ pole-mounted or projectile systems (typically fired from pneumatic rifles or crossbows) to biopsy sample or tag unrestrained animals, are most common. Remote biopsy sampling is an effective, mostly benign method of collecting fresh tissue samples from free-ranging cetaceans [1]. While cetaceans usually show some behavioural reaction to biopsy sampling, the reactions are typically mild and short-term (0.5–3 min) and the wounds made by the biopsy dart or punch heal quickly with no apparent adverse effects. Few studies, however, report on the behavioural and physiological impacts of remote biopsy sampling; this is important as different species and populations may react differently. No studies have shown long-term effects of biopsy sampling such as avoidance of the sampling area (e.g., [23]) or negative effects on reproduction and calf survival [24]; however, such effects are likely difficult to examine and only a small number of studies have attempted to do so [1].

Satellite tags are attached to animals using some form of sub-dermal retaining dart (e.g., [7], [25]). As with biopsy sampling, relatively few remote satellite (and earlier radio) tagging studies describe the behavioural reactions of animals to tagging – if they do it is largely qualitative – and mid- to long-term follow up studies are rare. The majority of immediate reactions to tagging seem to be unnoticeable or mild and short-term [25]–[29]. Best and Mate [30] found no major effect of satellite tagging on the reproductive success of adult female southern right whales *Eubalaena australis* or the survival of their calves. Tagging also does not appear to affect the survival or reproductive success of humpback whales *Megaptera novaeangliae* [29], [31].

One of the main challenges in remote satellite tagging systems is maximising the attachment durations of tags while minimising their invasiveness. Attachment durations have improved greatly (often hundreds of days currently compared to only a few days for the first attempts, see [25]) and tags have become smaller due to technological advances, but attachment duration remains highly variable. Remote satellite tagging studies were previously limited to large cetacean species, but the development of tags such as the ‘Low Impact Minimally Percutaneous External-electronics Transmitter’ configuration (LIMPET, [7]) has allowed tagging of smaller species such as killer whales *Orcinus orca*, Blainville’s beaked whales *Mesoplodon densirostris*, false killer whales *Pseudorca crassidens* and pygmy killer whales *Feresa attenuata* [7], [8], [32]–[34].

### Marion Island killer whales

Marion Island (46° 54′ S, 37° 45′ E), which lies in the Polar Frontal Zone in the Indian sector of the Southern Ocean, has a population of 58 identified killer whales which may occur at the island year round, but are most abundant between September and December [35], [36]. This population has been observed preying on southern elephant seals *Mirounga leonina*, sub-Antarctic fur seals *Arctocephalus tropicalis* and three penguin species, and the peak killer whale abundance coincides with the breeding seasons of these seals and penguins [35]. It is entirely unknown what proportion of the whales’ diet each species comprises and whether or not other prey (e.g., fishes, cephalopods) are taken, particularly when the whales are not observed at the island. Killer whales in the region depredate Patagonian toothfish *Dissostichus eleginoides* from longline fishing vessels [37], but it is unknown whether these individuals are from the Marion Island population or if toothfish are natural prey. When animals are not observed at the island their whereabouts and movements are unknown, although eight individuals have been photographically identified at both Marion Island and the Crozet Islands, located approximately 950 km east of Marion Island [36], [38]. The role of killer whales as drivers of seal and penguin population dynamics at Marion Island is important, but quantitatively uncertain [39]. The remoteness of Marion Island makes geographically wide-scale observations to elucidate diet and movement unfeasible and thus satellite tagging and biopsy sampling are vital methods to investigate the ecology of this population of killer whales.

### Aims

In this paper we, firstly, report our methods for shore-based, remote biopsy sampling and satellite tagging of killer whales, the success of these methods and particularly the attachment duration and performance of LIMPET satellite tags. Secondly, we describe the immediate behavioural reactions of animals to biopsy sampling and satellite tagging and test for differences in the reactions to each. Thirdly, we test whether researcher experience influences biopsy sampling and satellite tagging. Lastly, using multievent capture-recapture analysis, we evaluate whether biopsy sampling and satellite tagging changed the behaviour of individuals, altering mid- (1 month) and long-term (<24 months) sighting patterns.

## Methods

### Ethics statement

Biopsy sampling and tagging was approved by the University of Pretoria’s Animal Use and Care Committee (EC023-10) and the Prince Edward Islands Management Committee research and collection permits: 17/12; 1/2013; 1/2014.

### Field methods

**Figure 1:**
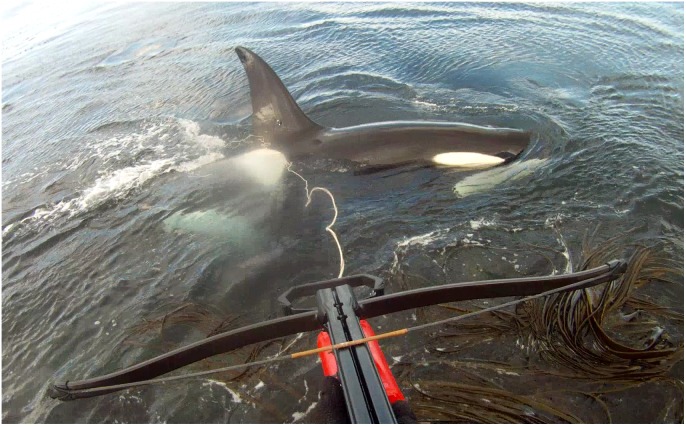
Satellite tagging of an adult male killer whale. Still frame from a point of view video showing satellite tagging of an adult male killer whale (M007) at Marion Island. The tag can be seen in the dorsal fin.

All killer whale studies at Marion Island are shore-based as boat-based work is not logistically possible or permitted [40]. Shore-based photographic identification (photo ID) has been successful at Marion Island as killer whales frequently approach within a few metres of the shore (Figure 1; [41]). This also allows work in weather conditions unsuitable for boat-based operations and importantly, in this study, allowed us to assess the reactions of animals to biopsy sampling and satellite tagging without any confounding reactions to boats.

We use ‘sampled’ and ‘sampling’ to refer to both biopsy sampling and satellite tagging; biopsy sampling is distinguished. We biopsy sampled and satellite tagged killer whales at two locations (Rockhopper Bay and Transvaal Cove) on the island’s leeward east coast, near (<1.0 km) a long-term observation/photo ID site [41]. Both locations are low rock ledges, 1.0–2.0 m above the water surface. Sampling attempts were made primarily during ‘dedicated observation sessions’, in which the marksman would wait for killer whales for a predetermined length of time (typically 3–10 hours). We used a 68 kg draw weight recurve crossbow (Barnett Panzer V; Barnett Outdoors, LLC, Tarpon Springs, Florida, United States of America) equipped with a red dot sight for biopsy sampling and satellite tagging. Bolts were tethered with line and a fishing reel mounted on the crossbow (Methods S1, [42]). Biopsy and tagging attempts were made by two arbalesters during the study and reactions – described in Table 1– were scored by the arbalester. After October 2011 the arbalester usually wore a high-definition video camera (GoPro HD Hero and GoPro HD Hero 2; Woodman Labs, Inc., Half Moon Bay, California, United States of America) to record biopsy and tagging attempts (Figure 1).

**Table 1:** Description of scores used to assess the immediate reactions of killer whales to biopsy sampling or tagging.

Score	Name	Description
0	None	No visible reaction
1	Slight	Slight flinch, slight shake, short acceleration, immediate submerge
2	Moderate	Pronounced flinch, pronounced shake, acceleration, prolonged dive
3	Strong	Prolonged dive and flight
4	Extreme	Breaching, tail slapping and flight (not observed in this study)

#### Biopsy sampling

We obtained tissue samples using stainless steel biopsy tips (25 mm×7 mm) attached to the bolts; a steel flange prevented penetration beyond 25 mm. Tips were sterilized before use and stored in clean plastic bags (Methods S1, [42]). The tissue samples obtained were stored for genetic, stable isotope and fatty acid analyses (Methods S1).

#### Satellite tagging

We deployed three models of satellite-linked telemetry devices: Sirtrack Kiwisat 202 (Sirtrack Ltd., Havelock North, New Zealand), Wildlife Computers SPOT5 and Wildlife Computers Mk10-A (Wildlife Computers, Redmond, Washington, United States of America). All three tag models allow estimation of geographic position via satellite using the Argos System (Collecte Localisation Satellites, Toulouse, France); the Mk10-A tag additionally includes a pressure (depth) sensor and a fast-response thermistor. Position estimates are classed by Collecte Localisation Satellites based on the estimated accuracy of the position, as follows: Class A and B – no estimate; 0– >1 500 m; 1–500-1 500 m; 2–250-500 m; 3– <250 m (Table S2) [43]. To extend tag battery life while maintaining biologically sensible data capture, tags were programmed with various transmission schedules or ‘duty cycles’ (Table S2).

**Figure 2:**
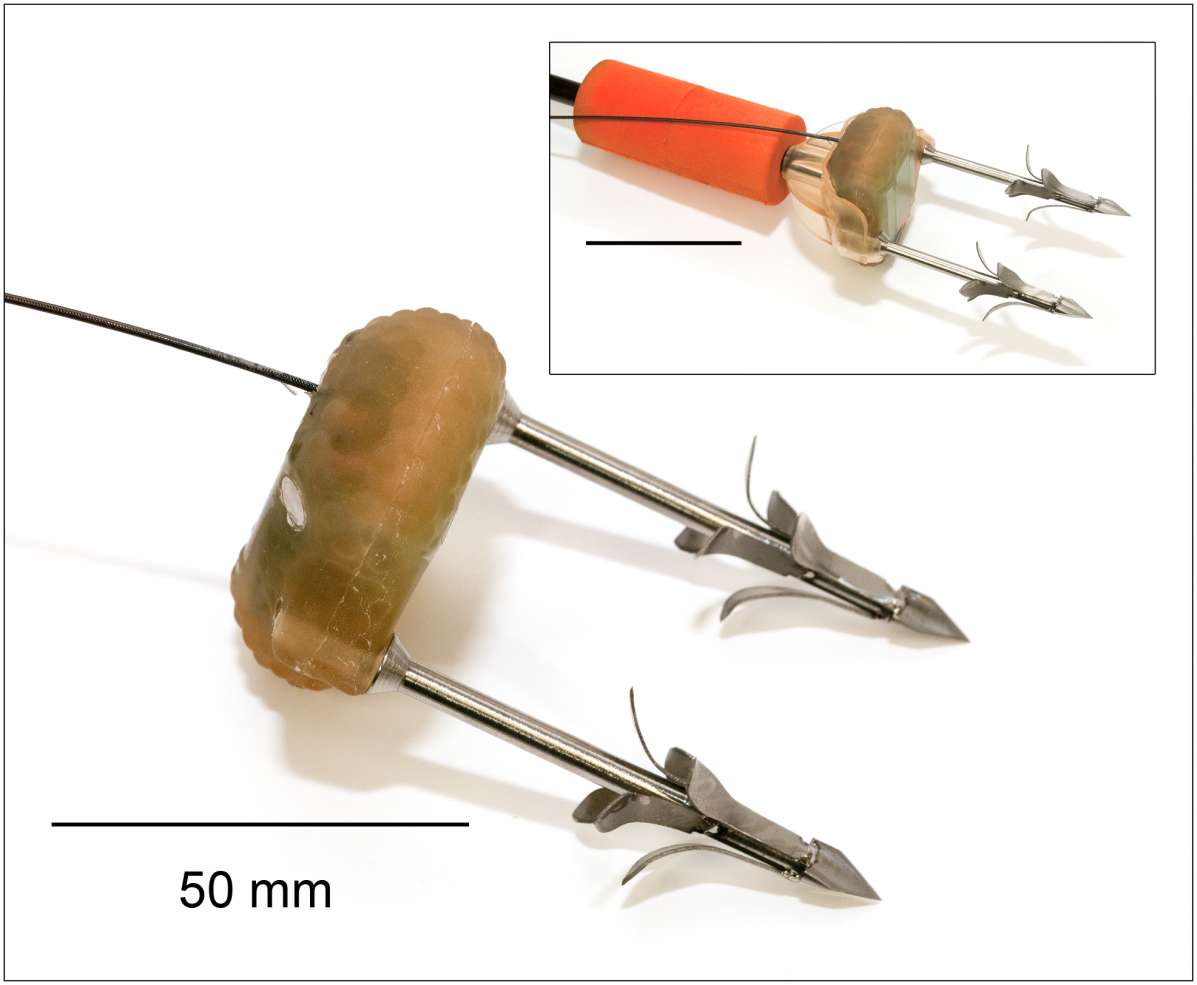
Wildlife Computers SPOT5 satellite-linked tag with attachment darts. The inset shows the tag in a deployment cup, attached to a crossbow bolt with float.

The tags were all in the LIMPET configuration where the tag is externally attached to the animal by sub-dermal darts which typically do not penetrate past the blubber layer (Figure 2; [7]). Penetration deeper than the length of the darts is prevented by the tag itself. This is in contrast to a typical ‘fully implantable’ tag where the transmitter is largely sub-dermal and the attachment darts (or anchors) may often penetrate through the blubber into the muscle (e.g., [25], [29]).

Kiwisat 202 tags were attached using 65 mm medical-grade stainless steel darts designed by RRR following [7]. Following an initial deployment with two darts (PTT 67764 in Table S2) we had difficulty attaching the tags and changed to a single dart design for these tags. SPOT5 and Mk10-A tags were attached using two 65 mm titanium darts designed and manufactured by RDA and Wildlife Computers (described in [7]). Tags (including darts) weighed 114 g (Kiwisat 202), 59 g (SPOT5) and 75 g (Mk10-A).

For deployment, tags were held on the crossbow bolt using urethane cups which fitted over the tag body (Figure 2). On impact with the animal, the sudden deceleration causes the tag to separate from the tag cup and bolt, which are retrieved using the tether (Figure 1; as for biopsy sampling). To prevent losing the tag if a shot was missed, Kiwisat 202 tags were additionally secured using two small screws which sheared the tag cup on impact with the animal and Wildlife Computer tags were secured using water soluble tape (which tore or dissolved) and monofilament tethers (which broke) on impact.

### Reactions to biopsy sampling and satellite tagging

We evaluated behavioural responses to tagging and biopsy by fitting generalized linear mixed models (GLMMs) using package lme4 in R [44], [45]. We treated reactions as binomial; i.e., no response vs. response. The reaction observations (n = 103) were not independent because we resampled some individuals and we therefore included *individual* as a random effect. Our candidate models included combinations of three variables which potentially affected response: *biopsy/tag* (whether a biopsy sampling or tagging attempt), *hit/miss* (whether the tag or biopsy arrow hit or missed the animal), and *class* (adult male, adult female or juvenile) (Table 2). Interactions between explanatory variables were not considered. Models were compared using Akaike's Information Criterion corrected for small sample sizes (AIC_c_). The model with the lowest AIC_c_ is the most parsimonious model in the model set [46].

**Table 2:** Model selection for the generalized linear mixed effects models (GLMMs) used to describe the reaction of killer whales to biopsy sampling and tagging.

Model	Np^a^	AIC_c_^b^	ΔAIC_c_^c^	*ω_i_* ^ d^
class + hit/miss	5	136.90	0.00	0.65
class + hit/miss + biopsy/tag	6	139.10	2.24	0.21
hit/miss	3	140.60	3.69	0.10
hit/miss + biopsy/tag	4	142.40	5.51	0.04
class	4	151.30	14.45	0.00
NULL	2	151.50	14.57	0.00
class + biopsy/tag	5	153.40	16.53	0.00
biopsy/tag	3	153.60	16.68	0.00

The full model was *reaction ∼class + hit/miss + biopsy/tag + (1|individual)*, where *reaction* was the response variable and *(1|individual)* denoted a random effect. All models included the random effect; only the predictor variables included in each model are shown.

Notes: ^a^number of parameters; ^b^small sample corrected Akaike Information Criterion; ^c^difference between the AIC_c_ score of the model in question and the best model; ^d^Akaike weight: relative likelihood of model in question divided by the sum of relative likelihoods for all models.

To test the validity of using binomial reactions rather than the reaction scores as defined in Table 1, we also compared the reaction scores using Kruskal-Wallis rank sum tests (kruskal.test in R) followed by multiple comparison tests where applicable (kruskalmc in package pgirmess in R; [47]).

### Effect of arbalester experience

To test whether the experience of an arbalester influenced the probability of hitting the target individual in a sampling event (*hit/miss*, as above), we fitted generalized linear models (GLMs) with a binomial error distribution in R. Both arbalesters were proficient marksmen and underwent training before fieldwork; however, neither had field experience of remote biopsy sampling or satellite tagging prior to this study. We therefore used the cumulative number of sampling attempts by the arbalester as a proxy for their experience level at each sampling attempt. Candidate models included all combinations of the following predictor variables: *experience*, *biopsy/tag* (as above), *arbalester* (the identity of the arbalester) and *range* (estimated range of the shot, in meters) (Table 3). As for the GLMMs, interactions between variables were not considered and AIC_c_ was used to compare models.

**Table 3:** Model selection for the generalized linear models (GL Ms) used to describe factors influencing the probability of hitting the target animal (*hit/miss*) during a sampling attempt.

Model	np^a^	AIC_c_^b^	ΔAIC_c_^c^	*ω_i_* ^ d^
range	2	140.50	0.00	0.24
experience + range	3	141.76	1.26	0.13
biopsy/tag + range	3	142.35	1.85	0.10
arbalester + range	3	142.60	2.10	0.09
NULL	1	142.99	2.50	0.07
experience + arbalester + range	4	143.47	2.97	0.06
biopsy/tag	2	143.52	3.02	0.05
experience + biopsy/tag + range	4	143.56	3.06	0.05
experience	2	144.29	3.79	0.04
biopsy/tag + arbalester + range	4	144.49	4.00	0.03
experience + biopsy/tag + range	3	144.62	4.12	0.03
arbalester	2	144.81	4.31	0.03
experience + arbalester	3	145.25	4.75	0.02
experience + biopsy/tag + arbalester + range	5	145.25	4.75	0.02
biopsy/tag + arbalester	3	145.44	4.95	0.02
experience + biopsy/tag + arbalester	4	145.63	5.13	0.02

The full model was *hit/miss ∼experience + biopsy/tag + range + arbalester*. Only the predictor variables included in each model are shown.

Notes: ^a^number of parameters; ^b^small sample corrected Akaike Information Criterion; ^c^difference between the AIC_c_ score of the model in question and the best model; ^d^Akaike weight: relative likelihood of model in question divided by the sum of relative likelihoods for all models.

### Sighting patterns

We used two approaches to detect changes in the sighting patterns of individuals after sampling using photographic identification sighting histories from 2006/04–2013/05 (sighting proportion) and 2008/05–2013/05 (mark-recapture). Briefly, dorsal fin photographs were taken during opportunistic (2006–2013) and dedicated (2008–2013) survey sightings and individuals were identified based on characteristic features such as scarring, mutilation and pigmentation. We stringently scored photographs based on their quality and used only good quality photographs to create a sighting history for each individual. All individuals were considered equally identifiable from good quality photographs, irrespective of the uniqueness of their characteristic features. Thus, individual variation in ‘recognisability’ should not affect the detection process (see [41] for methods). Sighting histories were restricted to sightings near (<1.0 km) the biopsy/tagging sites.

#### Sighting proportion

Firstly, following [23], we compared an individual’s ‘sighting proportion’ before and after sampling. For a given period, the sighting proportion was simply the number of photographic sightings of a given individual in that period divided by the number of photographic sightings of all individuals in that period. Sighting proportions were calculated for all sampled individuals before and after each sampling attempt and compared with a Wilcoxon paired Rank Sum Test (wilcox.test in R).

#### Mark-recapture analysis

Secondly, we used multievent mark-recapture models [48] to determine whether sampling reduced future detection probabilities. Typically, when individuals are physically captured, they may seek (trap-happy) or avoid (trap-shy) the sampling area (the ‘trap’) on future occasions [49]. We considered two possible responses to sampling. Firstly, sampling may result in temporary avoidance of sampling area, affecting detection only at the time-step following the one when the animal was sampled (‘trap-dependence’ in capture-recapture parlance [49], [50]). Alternatively, sampling may permanently alter individuals’ behaviour, resulting in a permanent state change with reduced detection following sampling, i.e., long-term trap-dependence. In this long-term trap-dependence model, instead of automatically returning to their initial state one time interval after being sampled [49], [50], individuals permanently remained in a ‘sampled’ state. For the purpose of our study, ‘normal’ trap-dependence corresponded to the mid-term (1 month) effect of sampling (Data S1), while long-term trap-dependence corresponded to the long-term (up to 24 months) effect of sampling (Data S2). Thus, in the model where response to sampling was temporary, animals reverted back to the naïve state after one month. Where sampling was assumed to permanently influence behaviour, the state change was permanent.

Before trying to estimate the effect of sampling on individuals’ behaviour, we had to account for intrinsic individual heterogeneity in detection, as failure to do so may lead to flawed inference [51]. One-sided directional test statistics (the signed square roots of the χ^2^-statistics) for Test3.SR (a test for transience) and Test2.CT (a test for trap-dependence) in U-CARE [52] suggested significant heterogeneity in detection (Table S3, [53] and references therein). We used capture-recapture mixture models [54], [55] that model heterogeneity using discrete ‘classes’ of individuals with low or high detection probability. Transience was accommodated by separately estimating the survival probability over the interval immediately following the first observation of the individual at Marion Island and survival during following intervals [56].

Mixture models specified the existence of two hidden states, representing individuals with distinct probabilities of detection. Our specification of two classes of individuals should not strictly be interpreted as evidence of the existence of two such classes; rather, these classes introduce heterogeneity in detection, improving model selection and reducing bias in parameter estimates [54].

Individual capture histories (n = 48) were based on photographic resightings between 2008 and 2013 (Data S1, Data S2). The full set of resightings for each individual was reduced to monthly ‘capture occasions’ (i.e., an individual was considered resighted or ‘captured’ in a month if it was photographed at least once in the month). At each occasion resighted individuals were known with certainty to be ‘sampled’ or ‘not sampled’. We thus defined three events: ‘not observed’, ‘resighted; not sampled’ and ‘resighted; sampled’. Depending on which of the above-described model structures we used, we defined up to nine states (Figure S2). Individuals moved in a Markovian way between the states. In the most complex model the states were thus: ‘Seen_t-1_; sampled’, ‘Not seen_t-1_; sampled’, ‘Seen_t-1_; not sampled’ and ‘Not seen_t-1_; not sampled’. Assigning the four states to two hidden groups with different detectability increased the number of states to eight. Finally, ‘death’ was explicitly included as a state. Transitions between states were decomposed as: 1) survival, 2) detection conditional on survival, and 3) sampling, given survival and detection (Figure S2). Models were fitted using program E-SURGE 1.9.0 [57].

Seasonality was introduced by separating the peak in killer whale abundance (September – December) from the rest of the year. Two periods of varying observer intensity (2008–2011 and 2011–2013) were also considered. Sampling was only possible when animals were seen, and sampling probabilities were constrained to the sampling period (2011–2013).

For both mid-term and long-term response to sampling, the same four initial candidate models were ranked using QAIC_c_ (sample size corrected, quasi-likelihood Akaike’s Information Criterion [46]). This initial set of four models was designed to help us decide on the best model structure for seasonality (winter/summer) among the following four options: 1) no seasonality; 2) same seasonality effect for all individuals; 3) seasonality applying to all individuals but in different strength for two hidden groups (suggesting variation in seasonal attendance between individuals); 4) seasonality applying only to one of the hidden groups (suggesting ‘residents’ and ‘migrants’). All models included two age classes for survival (transience model) and two periods of different field effort. They all included the effect of sampling (either long-term or mid-term). Having selected a seasonal model based on QAIC_c_, we removed the sampling effect from the model and evaluated the change in QAIC_c_.

## Results

**Figure 3:**
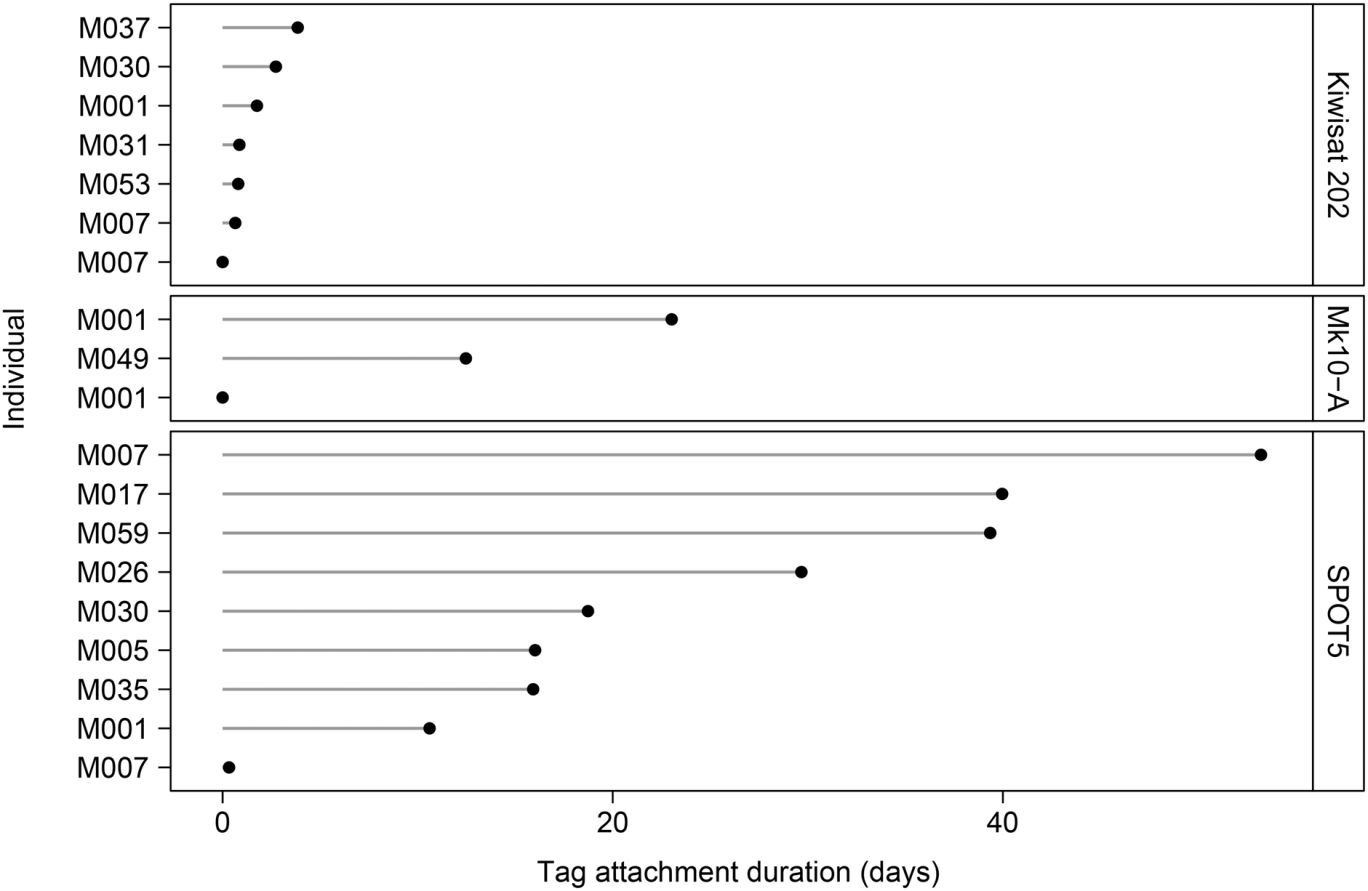
Attachment duration of satellite tags deployed on killer whales at Marion Island.

Overall, 109 biopsy and satellite tagging attempts were made, resulting in 71 hits (Table 4; Data S3). Of these, 101 attempts were made in 236 ‘dedicated observation sessions’ (on 231 days) totalling 1,645 hours – therefore an attempt was made every 16 h 17 m, overall. Biopsy hit rate was lower than tagging hit rates and biopsy sampling rate was low (43%). Tagging rate for Kiwisat 202 tags was very low (30%), reflecting–together with the short attachment durations (below and Figure 3)–the greater size and weight of these tags and the unsuccessful design of the attachment darts used with the tags. Tagging rate for the SPOT5 and Mk10-A tags was high (86%). Biopsy attempts were made at ranges from 3–20 m (average 8 m) and tagging attempts were made at ranges from 3–9 m (average 6 m).

**Table 4:** Number of biopsy sampling and satellite tagging attempts on killer whales at Marion Island.

	Attempts	Hits	Hitrate (%)^a^	Misses	Successfulhits^b^	Sampling/taggingrate (%)^a^
Biopsy	72	44	61.11	28	31	43.06
Tagging(Kiwisat 202)	23	15	65.22	8	7^c^	30.43
Tagging (SPOT5)	11	9	81.81	2	9^c^	81.81
Tagging (Mk10-A)	3	3	100.00	0	3^c^	100.00

Notes: ^a^Following [24]; ^b^Hit and tissue sample for biopsy sampling, hit and attach for satellite tagging; ^c^Tags attached, but did not necessarily penetrate properly.

### Satellite tags

We deployed 19 tags (Table S2). One Kiwisat 202 tag and 1 Mk10-A tag never transmitted. Both animals were resighted without tags 5 days later. Excluding these two instances, attachment duration was 0.6–3.9 days (Kiwisat 202), 0.3–53.2 days (SPOT5) and 12.5–23.0 days (Mk10-A) (Figure 3). Mean attachment duration (± SD) was 1.8±1.3 days, 24.9±16.8 days and 17.7±7.5 days, respectively. After taking duty cycle into account, the number of accurate position estimates (quality class 1–3) per transmission day (i.e., 24 transmission hours) was not significantly different between tag types (Kruskal Wallis χ^2^ = 2.21, *df* = 2, *p* = 0.33). Kiwisat 202 tags averaged (± SD) 10.7±3.0 accurate position estimates per transmission day while SPOT5s averaged 9.2±4.2 and Mk-10As averaged 12.0±2.8 accurate position estimates per transmission day (Table S2).

### Reactions to biopsy and satellite tagging attempts

**Figure 4:**
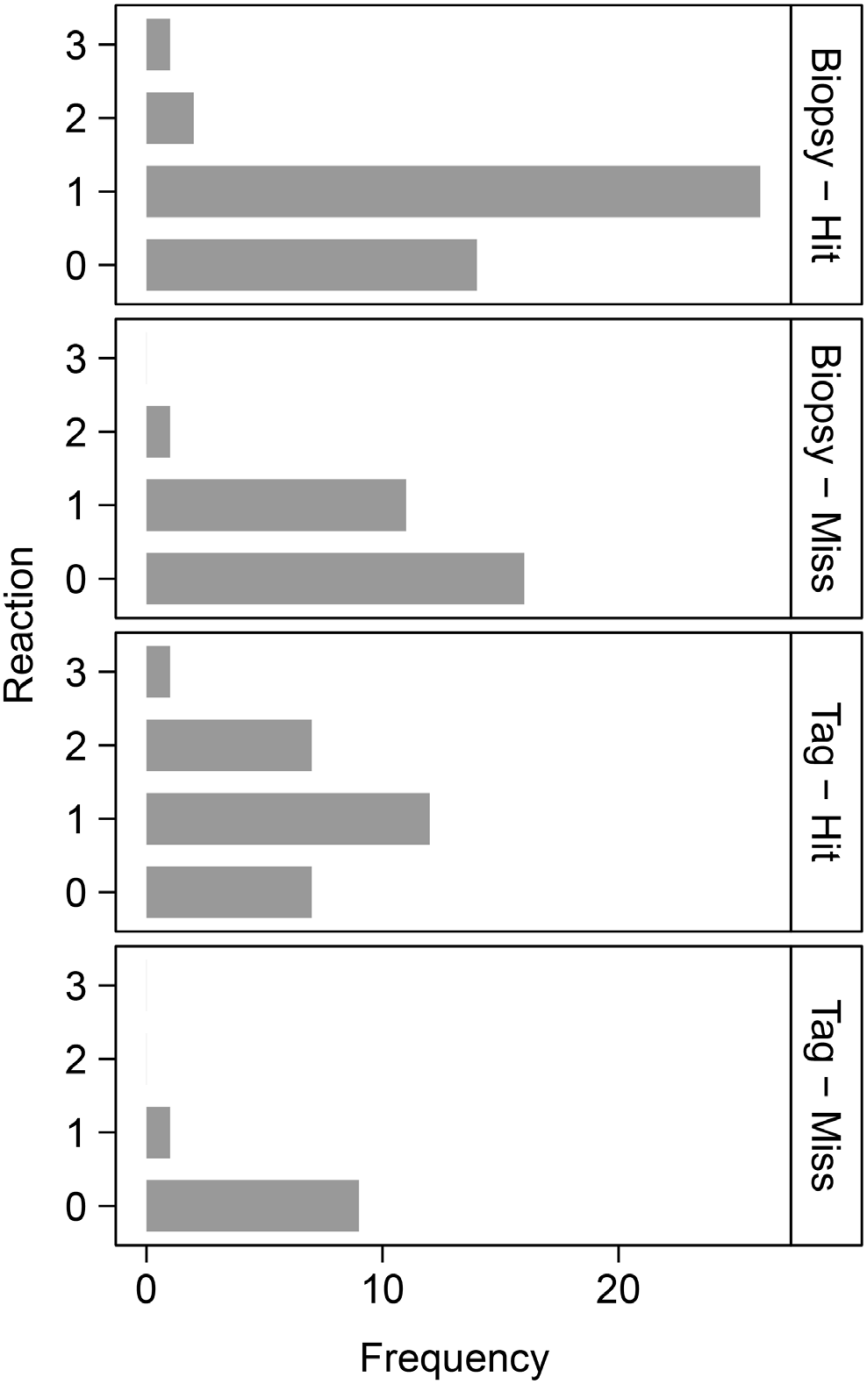
Immediate behavioural reactions to satellite tagging and biopsy sampling. Frequency of different immediate behavioural reactions of killer whales at Marion Island to tagging and biopsy sampling.

All responses corresponded to ‘no response’ and ‘low response’ in [1]. Several animals turned on their sides – they seemed to be looking at the arbalester, but may have been looking at the impact site (as described by [58]). Some animals rolled a number of times when tagged. Both such reactions were scored as 2 (Table 1); where the rolls were combined with an extended dive or flight the reactions were scored as 3. The most frequent reaction to a miss was 0 (no reaction), while the most frequent reaction to a hit was 1 (Figure 4). This was typically a slight acceleration, immediate submergence and/or a shake of the body (*cf.* [58]) (Table 1). Such responses were often so slight that they were difficult to see, even when reviewing video footage.

**Figure 5:**
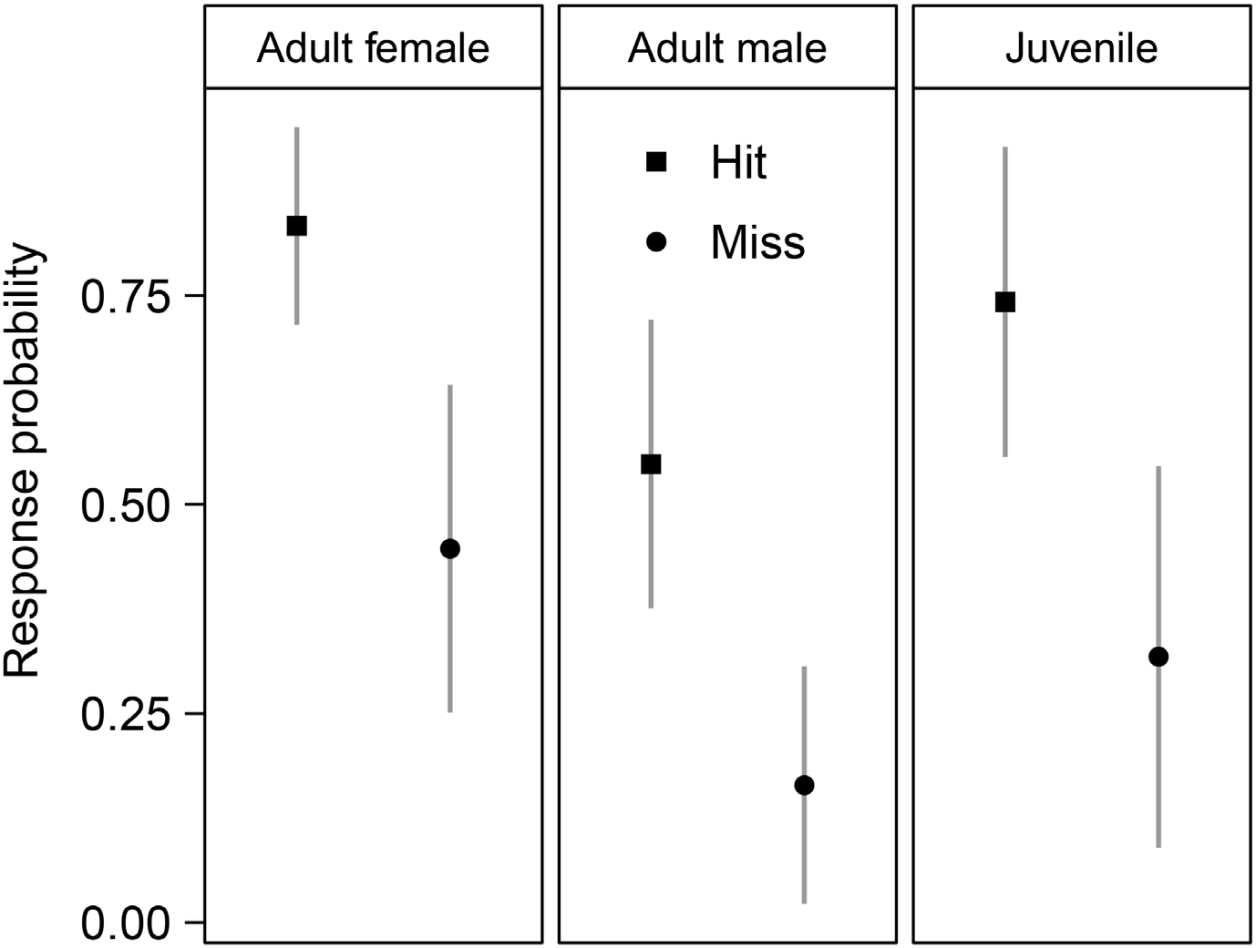
Predicted probability of an immediate behavioural response of killer whales to biopsy and tagging. Response probabilities as predicted by our best generalized linear mixed effects model, which included class (adult male, adult female or juvenile) and method (biopsy or tag); see Table 2.

In the GLMMs, the variance of the individual random effect was effectively zero, indicating either low individual variability in behavioural response, or that we were unable to detect individual variation with this limited data set. The model with the most support included *hit/miss* and *class* (adult male, adult female or juvenile) as predictor variables (Table 2). *Hit/miss* was the most important predictor variable (*ω_i_* = 1), followed by *class* (*ω_i_* = 0.86) (Table 2). *Biopsy/tag* had essentially no support, ranking lower than the null model when included as the only predictor variable. Adult females were most likely to respond, followed by juveniles and lastly males. Although the probability of response was highest when hit, behavioural responses were often present when missed (Figure 5).

Results of the Kruskal-Wallis tests support those of the GLMMs. Overall, there were significant response differences in the various categories (Kruskal-Wallis *χ^2^* = 18.48, *df* = 3, *p*<0.01). Reactions to tag and biopsy were not significantly different (*χ^2^* = 0.58, *df* = 1, *p* = 0.45) while reactions to hit and miss were (*χ^2^* = 13.812, *df* = 1, *p*<0.01). *Post-hoc* multiple comparisons showed significant differences between reactions to tag-hit and tag-miss, biopsy-hit and tag-miss, and biopsy-miss and tag-hit (Table S4).

### Effect of arbalester experience

The most supported model included only *range* as a predictor variable (β = −0.13±0.06, *p* = 0.038). Models including *experience* and *biopsy/tag* in addition to *range* had ΔAIC_c_<2, but only *range* was a significant or near-significant predictor in these models (Table 3).

### Sighting patterns

Changes in sighting proportion were typically small, and mean changes ranged from −0.02–0.68 percentage points (Figure S1). We found no significant differences when comparing sighting ratios before and after tagging/biopsy attempts; there also was no difference if we considered hits only (Table S1). The most frequently observed individual showed very large, positive changes in sighting proportion, but results remained the same if we repeated the comparison without this individual.

### Multievent mark-recapture

Models not accounting for heterogeneity performed poorly. The most parsimonious seasonality model allowed detection of both hidden groups to fluctuate independently with season. Removing seasonality from the one mixture group (thus creating a ‘resident’ group with constant detection throughout the year) increased the QAIC_c_ score.

**Figure 6:**
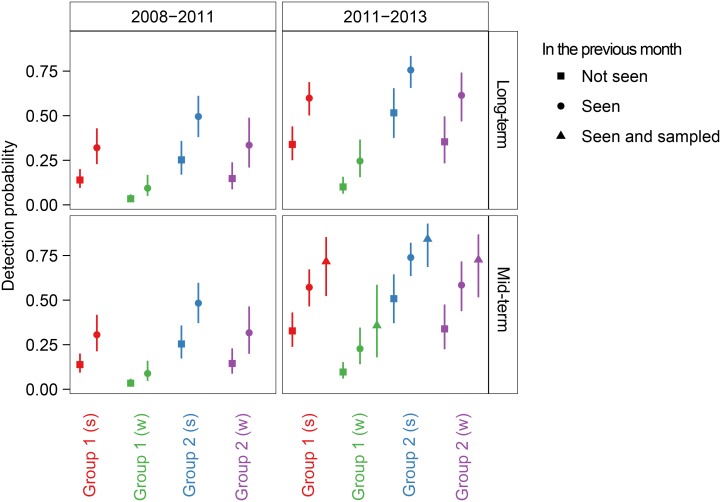
Detection probability of killer whales at Marion Island, given their capture history in the previous month. Detection probability (±95% confidence interval) was estimated using the highest ranked (lowest QAIC_c_) capture-recapture model in which sampling effect was assumed to be mid-term (1 month). Sampling effect was not in the highest ranked long-term (<24 months) model. The two time periods (2008–2011 and 2011–2013) correspond to different intensities of field effort; we only sampled in 2011–2013. ‘Groups’ refer to two classes of animals with distinct probabilities of detection (mixture components); *s* refers to the summer peak in killer whale abundance, *w* to the winter.

When sampling was modelled as a permanent state change, QAIC_c_ favoured removal of the sampling variable (Table 5). When sampling was modelled as a temporary state change, the sampling variable explained enough variation in detection probability to remain in the top ranked model, although the difference in QAIC_c_ was only 0.09, indicating that the effect of sampling on detection was weakly supported (Table 6). In that model, individuals seen and sampled during month *t*-1 had a higher probability of being detected in month *t* than individuals that were only seen (and not sampled) during month *t*-1 (Figure 6). Since we corrected for among-individual variation in sighting probability via the mixture model structure, this ‘trap-happy’ response suggests a possible bias towards sampling (and repeat-sampling) of ‘tamer’ individuals. Indeed, upon removing the individual that was most often seen and also repeatedly sampled and repeating the analysis, the model including sampling ranked lower than the model without the sampling effect (ΔQAIC_c_ = 1.13). Finally, the probability of sampling, given detection, was 0.18 (95% confidence interval: 0.14–0.25).

**Table 5:** Selection criteria for multievent capture recapture models of sighting histories of killer whales at Marion Island: long-term (up to 24 months) responses following sampling (tagging or biopsy) attempts.

Model	Np	Deviance	QAIC_c_^a^	ΔQAIC_c_	*ω_i_*
DH(2).season + trap + t_2008–2011;2011–2013_	10	1929.22	1122.23	0.00	0.58
DH(2).season + trap + t_2008–2011;2011–2013_+ sampling	11	1927.96	1123.60	1.37	0.29
DH(1).season + trap + t_2008–2011;2011–2013_+ sampling	10	1934.37	1125.17	2.94	0.13
season + trap + t_2008–2011;2011–2013_+ sampling	8	1982.16	1148.30	26.07	0.00
trap + t_2008–2011;2011–2013_+ sampling	7	2042.72	1180.83	58.60	0.00

‘Season’ refers to the same seasonality affect for all individuals. ‘DH(1).season’ refers to seasonality applying only to one of two hidden mixture groups (suggesting ‘resident’ and ‘migrant’ animals) while ‘DH(2).season’ refers to seasonality applying to all individuals but independently for two hidden groups (suggesting variation between individuals). ‘trap’ refers to a trap-dependence effect, ‘sampling’ refers to a sampling effect and ‘t_2008–2011;2011–2013_’ accounts for two periods with differing field effort.

Notes: ^a^ĉ = 1.75.

**Table 6:** Selection criteria for multievent capture recapture models of sighting histories of killer whales at Marion Island: mid-term (1 month) responses following sampling (tagging or biopsy) attempts.

Model	np	Deviance	QAIC_c_^a^	ΔQAIC_c_	*ω_i_*
DH(2).season + trap+ t_2008–2011;2011–2013_+ sampling	11	2088.03	1215.01	0.00	0.43
DH(2).season + trap + t_2008–2011;2011–2013_	10	2091.85	1215.10	0.09	0.41
DH(1).season + trap + t_2008–2011;2011–2013_+ sampling	10	2095.21	1217.02	2.01	0.16
season + trap + t_2008–2011;2011–2013_+ sampling	8	2143.99	1240.73	25.72	0.00
trap + t_2008–2011;2011–2013_+ sampling	7	2200.58	1270.98	55.97	0.00

‘Season’ refers to the same seasonality affect for all individuals. ‘DH(1).season’ refers to seasonality applying only to one of two hidden mixture groups (suggesting ‘resident’ and ‘migrant’ animals) while ‘DH(2).season’ refers to seasonality applying to all individuals but independently for two hidden groups (suggesting variation between individuals). ‘trap’ refers to a trap-dependence effect, ‘sampling’ refers to a sampling effect and ‘t_2008-2011;2011-2013_’ accounts for two periods with differing field effort.

Notes: ^a^ĉ = 1.75.

## Discussion

Our results suggests that land-based remote biopsy sampling and satellite tagging of killer whales at Marion Island are an effective means of collecting otherwise elusive data and the methods elicit only mild, short-term behavioural responses. We show the potential of multievent trap-dependence models (compared to simpler approaches such as [29]–[31]) to assess responses to sampling while controlling for intrinsic heterogeneity and other covariates. We found no mid- (1 month) or long-term (<24 months) avoidance of the study site following biopsy or tagging and conclude that there is no evidence of behavioural changes due to sampling.

### Biopsy sampling

Our successful biopsy sampling rate was low compared to biopsy sampling rates of odontocetes in other studies using bows (crossbows and compound bows) (mean ± SD = 68% ±19 percentage points in [1] compared to our 44%). Biopsy sampling rates of odontocetes with bows are typically lower than for mysticetes or using guns and poles [1], but we further attribute our low biopsy sampling rate to the tether line which worsens the crossbow’s already poor performance in wind (of which there is a great deal at Marion Island) and taking less than ideal shot opportunities as necessitated by the shore-based study. Although biopsy sampling opportunities are rare and required many hours of dedicated observations, shore-based work proved viable and we managed to biopsy sample nearly half of all identified whales in our population in the first two years of biopsy sampling. Biopsy sampling rates were lower than tagging rates mainly because tagging was only attempted at much closer ranges (3–9 m, mean = 6 m, compared with 3–20 m, mean = 8 m).

### Satellite tagging

Low tagging rates and short attachment durations meant that the Kiwisat 202 tags were not worth deploying (in a cost-benefit sense); this was due largely to poor attachment darts as the tags themselves performed well. The greater size and weight of that configuration probably contributed to their short attachment times – larger tags are subject to greater drag in the water and heavier tags slow the bolt’s speed when fired, which may mean that darts do not consistently penetrate to their full depth. This also affected the trajectory of the shot – the heavier tags did not always strike at an appropriate angle, necessitating a single-dart design which further reduced attachment duration. This underlines the importance of using proven techniques and technologies in biopsy and tagging studies. When these are not available, methods and equipment should be developed with the input of those with relevant expertise and experience (e.g., field biologists, engineers, veterinarians) and tested in as realistic a way as possible (e.g., using cetacean carcasses to test tagging and biopsy techniques [59]). When species or populations of special conservation concern are involved, methods and equipment may need to be tested on other species or populations first [12].

Attachment durations were longer but highly variable (like other studies report) for SPOT5 and Mk10-A tags and still short compared to fully implantable tags (e.g., [60], [61]). This represents the compromise of a minimally invasive, external tag attachment which can be deployed on smaller species compared to configurations where the tag itself is fully implanted, as used on large whales. Our average SPOT5 and Mk10-A deployment durations were shorter than, but as variable as, other studies using the same tag setup (mean ± SD = 24±24 d in [7]; 43±23 d in [32], 32±22 d in [8] and 46±41 d in [34]). At Marion Island killer whales frequently hunt and patrol in dense bull kelp *Durvillaea antarctica* and giant kelp *Macrocystis pyrifera* forests which circle the island inshore, and we suggest that this may shorten attachment durations as tags may become ensnared. We obtained a greater number of accurate position estimates per day than large whale studies using fully implantable tags (e.g., 1.5±1 in [6], 2±1.6 in [61]), but we anticipated shorter deployments than those studies and our tags were programmed to transmit more frequently. Killer whales also have shorter dive durations than large whales. The LIMPET setup is thus currently more useful for finer scale movement studies.

### Reactions

Reactions to tagging were similar to the few responses described in other tagging studies [7], [25], [26], [29], [62] and to reactions in other biopsy studies (reviewed by [1]), although there were no ‘strong’ (*sensu* [1]) reactions in our study. Some authors have attributed responses largely to the research boat rather than the actual tagging or biopsy, but we show that killer whales do respond to shore-based tagging and biopsy (as in [7]).

Although slightly stronger reactions were more frequent in response to tagging, the type of sampling (biopsy sampling or tagging) was not important in determining whether an animal would respond. Similarly, Reeb and Best [63] noted that southern right whales’ reactions do not differ when biopsied with deep (11–20.5 cm) darts compared to more superficial darts used in a previous study [24]. This might suggest that, in general, responses to biopsy sampling and tagging are primarily startle, and not pain, responses. However in our study hit *vs*. miss did influence reactions, indicating that there is an effect of an object hitting the animal’s body compared to hitting the water. We cannot say whether hitting the animal’s body is simply more startling to the animal or if, and how much, pain plays a role.

Some individual variation in behavioural reactions may be expected, but this was not evident in our study. It is possible that our data were too few to detect consistent individual variation. Sex and age, however, did influence reactions. Adult males were less likely to react than juveniles and adult females. Other studies report that group composition influences reaction but very few studies report sex-differences: Brown et al. [64] reported that female humpback whales responded more often to biopsy sampling, Gauthier and Sears [65] report the same for female fin whales *Balaenoptera physalus*.

### Effect of arbalester experience

Noren and Mocklin [1] name research team experience as an important factor influencing the success of collecting biopsy samples from cetaceans (although only [58] provides any qualitative support for the statement). We found almost no support for an effect of arbalester experience on sampling success, however such an effect may be obscured by the baseline proficiency of the arbalesters (both had undergone training prior to fieldwork), may only become apparent after even more experience (e.g., hundreds of sampling attempts compared to less than one hundred in this study), or may be stronger in vessel-based studies, where the vessel driver’s experience is also relevant (e.g., [58]). Regardless, research team experience remains an important consideration in terms of animal welfare. Consequences of inaccurate shooting may include: hitting non-target animals; hitting target animals at the wrong body location - an important concern for satellite tags which need to be above water to transmit and for biopsy samples where tissue characteristics may vary, affecting subsequent analyses [3]; and the loss of equipment. Hitting a non-target animal or the wrong place on the body may result in serious injury to the animal.

### Sighting rates

Multievent models provided a flexible framework to model the response of individuals to sampling while accounting for demographic processes of the population. The sighting ratio method assumed that ‘all animals are equal’ with regards to seasonal movement and thus availability for detection; this heterogeneity could confound the results of a simple analysis. In this study the results were not fundamentally different: neither demonstrated a negative response to tagging or biopsy. However, the multievent approach showed the important effect of seasonal occurrence and different residence patterns which influenced sighting probabilities. The weak mid-term (∼1 month) positive response to sampling seemed to be caused by a single individual, which underlines the importance of taking individual variation in sighting rates into account. This also highlights potential sampling biases (e.g., sex-biased biopsy sampling [66]) which we could fortunately detect by photographic identification of all sampled individuals. Individuals that centre their home ranges in the study area and have higher sighting rates are more likely to be sampled due to their general availability. Field effort will need to continue in order to generate enough chances to sample animals that occasionally visit the sampling area.

### Can sampling lead to mid- or long-term behavioural changes?

Whether or not biopsy sampling and satellite tagging can lead to mid or long-term changes in behaviour depends on several factors. Firstly, an individual must be aware of the sampling attempt. We have shown that individuals do react to sampling attempts (58% of attempts), and are thus often aware of them. However, the absence of a visible behavioural response to a sampling attempt does not necessarily imply that the animal is unaware of the attempt. Several studies have shown physiological responses to human disturbance where there was little or no behavioural response (e.g., [67]–[69]). This underlines the utility of measuring physiological stress indicators such as glucocorticoid hormones or heart rate, however in many cases such measurement itself will result in stress, confounding the measurements [70], [71]. Secondly, the sampling attempt must be perceived negatively by the individual. We assume the immediate behavioural reactions sometimes associated with biopsy sampling - such as defecation, tail slapping, breaching and flight from the area - (see Table 3 in [1]) indicate a negative stimulus, be it fright or pain. Thirdly, in our case where sampling attempts were land-based at two locations, the individual must be able to associate its experience (the sampling attempt) with a spatial location or other cue (seeing the arbalester, for example) and this memory must persist for some length of time. This would seem well within the capabilities of many animals (e.g., [72]–[74]) and certainly killer whales, which range widely but show strong interannual site fidelity (at Marion Island - [41]) and are cognitively complex [75]. Lastly, given the above, the strength of the negative experience must be sufficient to alter behaviour. Animals may not show a mid-term behavioural response because the motivation to perform an activity (e.g., foraging), or to remain at a high quality site, may exceed the motivation to avoid sampling; individuals may also lack suitable habitat to disperse to in order to avoid sampling. This can be framed as a cost-benefit tradeoff if the disturbance stimulus (in this case sampling) is equated to predation or injury risk [76], [77]. This may beg the question whether killer whales - which do not have significant natural predators - are less sensitive to disturbance stimuli.

Our two sampling locations, <1 km apart, represent a short stretch of the ∼50 km stretch of Marion Island coastline patrolled by killer whales [41], [78], [79]. Breeding colonies of killer whale prey (seals and penguins) at these sites represent a small proportion of the total breeding populations of these species at Marion Island (Table S5). We consider it plausible that an individual killer whale could alter its path by a few hundred meters to avoid the sampling sites, and that this would not represent a considerable energy cost or loss of foraging opportunity. Social bonds may possibly prevent sampling site avoidance, particularly when only some group members have been sampled, but our analyses of the social structure of Marion Island killer whales over 7 years (RRR and PJNdB, in preparation) indicates considerable flexibility in social groups. Half Weight Association Index values – an estimate of the proportion of time two animals spend together – range from 0.21–0.66 (average ± SD = 0.48±0.18) within defined social units, clearly indicating that animals are not constantly associated. Further, 370 (13%) of 2,821 sightings recorded in that study were of single (lone) individuals. This suggests that social bonds between killer whales will not necessarily prevent individuals from avoiding the sampling sites.

The factors we have mentioned which may prevent short term disturbance (sampling) from causing mid-term behavioural changes are intractable in this study, but could stimulate further research in different species or settings. There is debate as to how well behavioural changes signal the sensitivity of animals to disturbance [80]. In cetaceans, documented disturbance is likely largely due to direct or associated noise (e.g., [81] for killer whales). The mid- to long-term sensitivity of cetaceans to satellite tagging and biopsy sampling is unknown, but seems negligible. Best et al. [24] show sensitization to biopsy sampling up to 65 days in female southern right whales with calves, but such cases seem rare [1].

Importantly, we found no significant long-term (<24 months) changes in the sighting probability of tagged or biopsied killer whales. In the only study using a comparable method to ours, Tezanos-Pinto and Baker [23] found no difference in the long-term sighting probabilities between biopsied and non-biopsied bottlenose dolphins *Tursiops truncatus*. Our study supports the idea that cetaceans do not change their long-term behaviour in response to being sampled. However, if such responses are subtle, they may require considerable data and time to detect. We have not tested for physiological responses (e.g., stress) on any temporal scale, nor for an impact on hunting behaviour and demographic performance.

However, one of our stated aims was to ‘evaluate whether biopsy sampling and satellite tagging changed the behaviour of individuals, altering mid- (1 month) and long-term (<24 months) sighting patterns.’ We wished to evaluate any behavioural changes to our tagging and biopsy sampling protocol, rather than determine the mechanisms affecting such behavioural changes (or lack thereof, as we found). Our results are therefore meaningful independent of any evaluation of intermediate factors, however we recommend longer term monitoring to assess whether satellite tagging and biopsy sampling have any effect on demographic parameters (e.g., [82]).

## Conclusions

Remote biopsy sampling and satellite tagging of killer whales from shore is successful at Marion Island and these methods can provide insights into the ecology of this population which is difficult to access at sea. We found that reactions to biopsy sampling and satellite tagging were mild or unnoticeable and we found no significant mid- or long-term changes in the occurrence of killer whales at the study site. However, long-term monitoring of individuals after biopsy sampling and tagging should continue in order to provide continuous assessment of potential impacts on the study animals. Such monitoring should be implemented in other studies where animals are biopsied or tagged, especially considering the increased use of these methods.

## Supporting Information

Figure S1 **Changes (percentage points) in the sighting proportion of killer whales at Marion Island following various sampling events.** a) tag or biopsy – first attempt; b) biopsy – first attempt; c) biopsy – first hit; d) tag – first attempt; e) tag – first hit. Sighting proportion (%) was calculated as the number of sightings of an individual during a given period, divided by the number of sightings of all individuals in the same period. Negative change thus indicates an individual was seen less following a sampling event.
(TIF)

Figure S2 **A multinomial tree diagram with arrows denoting the possible transitions between states (solid boxes) from** ***t*** **to** ***t+1*** **.** States occupied are not directly observed, but events (dashed boxes) represent observations following initial capture (‘Encounter’). Individuals belong to one of two hidden classes with distinct probabilities of detection; movement between detection groups over time is not allowed. Entry to the population conditions on the first encounter (‘Seen’) and all individuals are seen once or more prior to sampling (‘Initial state’ step). Subsequent state transition probabilities are decomposed in three steps as the product of the probabilities of ‘Survival’, ‘Detection’ and ‘Sampling’. Only individuals that are detected (‘Seen’) can be sampled. Once sampled, individuals either remain in the sampled state (permanent state change scenario; solid arrows) or may move back to the ‘Not Sampled’ state at the next occasion (mid-term sampling effect scenario; dashed arrows).
(TIF)

Table S1 **Comparisons of sighting proportions before and after tagging and biopsy attempts on killer whales at Marion Island (paired Wilcox rank sum test).** The sighting proportion is the number of photographic sightings of an individual in a given period, divided by the number of photographic sightings of all individuals in that period (following [1]). Notes: ^a^ *N* is the number of sampling attempts included for each comparison. ^b^ *W* is the test statistic. ^c^ *Tag or biopsy – first attempt* includes only the first attempt (regardless of whether it was a tag or biopsy attempt), hence it is not the sum of *Tag – first attempt* and *Biopsy – first attempt*.
(DOCX)

Table S2 **Satellite tags deployed on killer whales at Marion Island showing the attachment duration, duty cycle and number of position estimates received.** Notes: ^a^SA – subadult, A – adult; ^b^1– transmit 00∶00–24∶00 UTC, 2– transmit 00∶00–06∶00 and 12∶00–18∶00 UTC, 3– transmit 01∶00–22∶00 UTC for 30 days, thereafter 01∶00–22∶00 UTC on every second day, 4– transmit 01∶00–22∶00 UTC for 25 days, thereafter 01∶00–22∶00 UTC on every fourth day; ^c^Argos position estimate quality class (see text for accuracy); ^d^‘Accurate’ position estimates are quality class 1–3; number of accurate positions estimates per day was corrected for duty cycle (the proportion of time transmitting) and is thus expressed per ‘transmission day’, i.e., 24 transmission hours.
(DOCX)

Table S3 **Approximate goodness of fit (GOF) tests for individual capture histories of killer whales at Marion Island.** The overdispersion coefficient (*ĉ*) for a heterogeneity model including transience and trap-happiness was computed by removing the squared directional test statistics from the time dependant model [1].
(DOCX)

Table S4 **Multiple comparisons test (kruskalmc in R package pgirmess** [1] **) results for significant reaction differences to tagging and biopsy attempts of various types.**
(DOCX)

Table S5 **Breeding populations of known killer whale prey at satellite tagging and biopsy sampling locations, and total breeding populations, at Marion Island.** Seal numbers refer to pup production and penguin numbers to breeding pairs. Numbers in parentheses are percentage of the total breeding population. Dashes indicate zero animals.
(DOCX)

Data S1 **Encounter history matrix of killer whales at Marion Island, with temporary state change**. Monthly encounter history matrix (May 2008–May 2013) of 48 killer whales. States are indicated as: 0– not seen and not sampled; 1– seen but not sampled; 2– seen and sampled. The sampled state is not permanent (i.e., individuals return to an unsampled state after 1 month).
(CSV)

Data S2 **Encounter history matrix of killer whales at Marion Island, with permanent state change.** Monthly encounter history matrix (May 2008–May 2013) of 48 killer whales. States are indicated as: 0– not seen and not sampled; 1– seen but not sampled; 2– seen and sampled. The sampled state is permanent (i.e., individuals subsequently remain in the sampled state, if seen).
(CSV)

Data S3 **Satellite tagging and biopsy sampling of killer whales at Marion Island.** Satellite tagging and biopsy sampling attempts are shown, with associated data. Class: AM – adult male; AF – adult female; J – juvenile. Success: Y – yes (hit and sample for biopsy sampling attempts, hit and attach for satellite tagging attempts); N – no. Reaction: see Table S1 in text. Range – range of the attempt (in meters). Attempt – cumulative attempts by the arbalester.
(CSV)

Methods S1 **Further information about field methods used.**
(DOCX)

## References

[1] Noren DP, Mocklin JA (2012) Review of cetacean biopsy techniques: factors contributing to successful sample collection and physiological and behavioral impacts. Mar Mammal Sci 28: 154–199 10.1111/j.1748-7692.2011.00469.x

[2] Hoelzel AR, Dahlheim ME, Stern SJ (1998) Low genetic variation among killer whales (*Orcinus orca*) in the Eastern North Pacific and genetic differentiation between foraging specialists. J Hered 89: 121–128. 9542159 10.1093/jhered/89.2.121

[3] Budge SM, Iverson SJ, Koopman HN (2006) Studying trophic ecology in marine ecosystems using fatty acids: a primer on analysis and interpretation. Mar Mammal Sci 22: 759–801.

[4] Newsome SD, Clementz MT, Koch PL (2010) Using stable isotope biogeochemistry to study marine mammal ecology. Mar Mammal Sci 26: 509–572 10.1111/j.1748-7692.2009.00354.x

[5] Hunt KE, Moore MJ, Rolland RM, Kellar NM, Hall AJ, et al.. (2013) Overcoming the challenges of studying conservation physiology in large whales: a review of available methods. Conserv Physiol 1. doi:10.1093/conphys/cot006. 10.1093/conphys/cot006 PMC4806609 27293590

[6] Baumgartner MF, Mate BR (2005) Summer and fall habitat of North Atlantic right whales (*Eubalaena glacialis*) inferred from satellite telemetry. Can J Fish Aquat Sci 62: 527–543 10.1139/f04-238

[7] Andrews RD, Pitman RL, Ballance LT (2008) Satellite tracking reveals distinct movement patterns for Type B and Type C killer whales in the southern Ross Sea, Antarctica. Polar Biol 31: 1461–1468.

[8] Baird RW, Schorr GS, Webster DL, McSweeney DJ, Hanson MB, et al (2010) Movements and habitat use of satellite-tagged false killer whales around the main Hawaiian Islands. Endanger Species Res 10: 107–121 10.3354/esr00258

[9] Bilgmann K, Möller LM, Harcourt RG, Gales R, Beheregaray LB (2008) Common dolphins subject to fisheries impacts in Southern Australia are genetically differentiated: implications for conservation. Anim Conserv 11: 518–528 10.1111/j.1469-1795.2008.00213.x

[10] Maxwell SM, Hazen EL, Bograd SJ, Halpern BS, Breed GA, et al (2013) Cumulative human impacts on marine predators. Nat Commun 4: 1–9 10.1038/ncommsS3688 24162104

[11] Wilson R, McMahon C (2006) Measuring devices on wild animals: what constitutes acceptable practice? Front Ecol Environ 4: 147–154.

[12] Cooke SJ (2008) Biotelemetry and biologging in endangered species research and animal conservation: relevance to regional, national, and IUCN Red List threat assessments. Endanger Species Res 4: 165–185 10.3354/esr00063

[13] Wilson RP, Kreye JM, Lucke K, Urquhart H (2004) Antennae on transmitters on penguins: balancing energy budgets on the high wire. J Exp Biol 207: 2649–2662 10.1242/jeb.01067 15201297

[14] Hazekamp AAH, Mayer R, Osinga N (2010) Flow simulation along a seal: the impact of an external device. Eur J Wildl Res 56: 131–140 10.1007/s10344-009-0293-0

[15] Saraux C, Le Bohec C, Durant JM, Viblanc VA, Gauthier-Clerc M, et al (2011) Reliability of flipper-banded penguins as indicators of climate change. Nature 469: 203–206. 21228875 10.1038/nature09630

[16] Bateson P (1986) When to experiment on animals. New Sci 109: 30–32. 11655736

[17] McMahon CR, Harcourt RG, Bateson P, Hindell MA (2012) Animal welfare and decision making in wildlife research. Biol Conserv 153: 254–256 10.1016/j.biocon.2012.05.004

[18] Gales NJ, Bowen WD, Johnston DW, Kovacs KM, Littnan CL, et al (2009) Guidelines for the treatment of marine mammals in field research. Mar Mammal Sci 25: 725–736 10.1111/j.1748-7692.2008.00279.x

[19] Field IC, Harcourt RG, Boehme L, de Bruyn PJN, Charrassin J-B, et al (2012) Refining instrument attachment on phocid seals. Mar Mammal Sci 28: E325–E332 10.1111/j.1748-7692.2011.00519.x

[20] McMahon CR, Hindell MA, Harcourt RG (2013) Publish or perish: why it’s important to publicise how, and if, research activities affect animals. Wildl Res 39: 375–377 10.1071/WR12014

[21] Wells RS, Rhinehart HL, Hansen LJ, Sweeney JC, Townsend FI, et al (2004) Bottlenose dolphins as marine ecosystem sentinels: developing a health monitoring system. Ecohealth 1: 246–254.

[22] Elwen SH, Meyer MA, Best PB, Kotze PGH, Thornton M, et al (2006) Range and movements of female Heaviside’s dolphins (*Cephalorhynchus heavisidii*), as determined by satellite-linked telemetry. J Mammal 87: 866–877.

[23] Tezanos-Pinto G, Baker C (2012) Short-term reactions and long-term responses of bottlenose dolphins (*Tursiops truncatus*) to remote biopsy sampling. New Zeal J Mar Freshw Res 46: 13–29 10.1080/00288330.2011.583256

[24] Best PB, Reeb D, Rew MB, Palsbøll PJ, Schaeff C (2005) Biopsying southern right whales: their reactions and effects on reproduction. J Wildl Manage 69: 1171–1180.

[25] Mate B, Mesecar R, Lagerquist B (2007) The evolution of satellite-monitored radio tags for large whales: one laboratory’s experience. Deep Sea Res Part II Top Stud Oceanogr 54: 224–247 10.1016/j.dsr2.2006.11.021

[26] Watkins WA (1981) Reaction of three species of whales *Balaenoptera physalus*, *Megaptera novaeangliae,* and *Balaenoptera edeni* to implanted radio tags. Deep Sea Res Part A Oceanogr Res Pap 28: 589–599 10.1016/0198-0149(81)90119-9

[27] Watkins WA, Tyack P (1991) Reaction of sperm whale (*Physeter catodon*) to tagging with implanted sonar transponder and radio tags. Mar Mammal Sci 7: 409–413.

[28] Goodyear JD (1993) A sonic/radio tag for monitoring dive depths and underwater movements of whales. J Wildl Manage 57: 503–513.

[29] Robbins J, Zerbini AN, Gales N, Gulland FMD, Double M, et al.. (2013) Satellite tag effectiveness and impacts on large whales: preliminary results of a case study with Gulf of Maine humpback whales. Report SC/65a/SH05 presented to the International Whaling Commission Scientific Committee, Jeju, Korea.

[30] Best PB, Mate B (2007) Sighting history and observations of southern right whales following satellite tagging off South Africa. J Cetacean Res Manag 9: 111–114.

[31] Mizroch SA, Tillman MF, Jurasz S, Straley JM, Von Ziegesar O, et al (2011) Long-term survival of humpback whales radio-tagged in Alaska from 1976 through 1978. Mar Mammal Sci 27: 217–229 10.1111/j.1748-7692.2010.00391.x

[32] Schorr GS, Baird RW, Hanson MB, Webster DL, McSweeney DJ, et al (2009) Movements of satellite-tagged Blainville’s beaked whales off the island of Hawai’i. Endanger Species Res 10: 203–213 10.3354/esr00229

[33] Baird RW, Schorr GS, Webster DL, McSweeney DJ, Hanson MB, et al (2011) Movements of two satellite-tagged pygmy killer whales (*Feresa attenuata*) off the island of Hawai’i. Mar Mammal Sci 27: E332–E337 10.1111/j.1748-7692.2010.00458.x

[34] Durban JW, Pitman RL (2012) Antarctic killer whales make rapid, round-trip movements to subtropical waters: evidence for physiological maintenance migrations? Biol Lett 8: 274–277 10.1098/rsbl.2011.0875 22031725 PMC3297399

[35] Reisinger RR, de Bruyn PJN, Tosh CA, Oosthuizen WC, Mufanadzo NT, et al (2011) Prey and seasonal abundance of killer whales at sub-Antarctic Marion Island. African J Mar Sci 33: 99–105 10.2989/1814232X.2011.572356

[36] Reisinger RR, de Bruyn PJN (2014) Marion Island killer whales: 2006–2013. Mammal Research Institute, University of Pretoria. doi:10.6084/m9.figshare.971317.

[37] Williams AJ, Petersen SL, Goren M, Watkins BP (2009) Sightings of killer whales *Orcinus orca* from longline vessels in South African waters, and consideration of the regional conservation status. African J Mar Sci 31: 81–86 10.2989/AJMS.2009.31.1.7.778

[38] Tixier P, Gasco N, Guinet C (2014) Killer whales of the Crozet Islands: photo-identification catalogue 2014. Villiers en Bois: Centre d’Etudes Biologiques de Chizé - CNRS. doi:10.6084/m9.figshare.1060247.

[39] Reisinger RR, de Bruyn PJN, Bester MN (2011) Predatory impact of killer whales on pinniped and penguin populations at the Subantarctic Prince Edward Islands: fact and fiction. J Zool 285: 1–10 10.1111/j.1469-7998.2011.00815.x

[40] Prince Edward Islands Management Plan Working Group (1996) Prince Edward Islands Management Plan. Pretoria: Department of Environmental Affaris and Tourism.

[41] Reisinger RR, de Bruyn PJN, Bester MN (2011) Abundance estimates of killer whales at subantarctic Marion Island. Aquat Biol 12: 177–185 10.3354/ab00340

[42] Lambertsen RH (1987) A biopsy system for large whales and its use for cytogenetics. J Mammal 68: 443–445 10.2307/1381495

[43] Collecte Localisation Satellites (2011) Argos User’s Manual. Toulouse: Collecte Localisation Satellites.

[44] Bates D, Maechler M, Bolker B, Walker S (2013) lme4: Linear mixed-effects models using Eigen and S4. R package version 1.0–4.

[45] R Development Core Team (2013) R: A language and environment for statistical computing. Vienna, Austria: R Foundation for Statistical Computing.

[46] Burnham KP, Anderson DR (2002) Model selection and multimodel inference: a practical information-theoretic approach. Second edi. New York: Springer.

[47] Giraudoux P (2011) pgirmess: Data analysis in ecology. R package version 1.5.1. http://cran.r-project.org/web/packages/pgirmess/index.html.

[48] Pradel R (2005) Multievent: an extension of multistate capture recapture models to uncertain states. Biometrics 61: 442–447. 16011690 10.1111/j.1541-0420.2005.00318.x

[49] Pradel R, Sanz-Aguilar A (2012) Modeling trap-awareness and related phenomena in capture-recapture studies. PLoS One 7: e32666 10.1371/journal.pone.0032666 22396787 PMC3292565

[50] Pradel R (1993) Flexibility in survival analysis from recapture data: handling trap-dependence. In: Lebreton JD, North PM, editors. Marked individuals in the study of bird populations. Basel: Birkhauser Verlag. 29–37.

[51] Lebreton JD, Burnham KP, Clobert J, Anderson DR (1992) Modelling survival and testing biological hypotheses using marked animals: a unified approach with case studies. Ecol Monogr 62: 67–118.

[52] Choquet R, Lebreton JD, Gimenez O, Reboulet A-M, Pradel R (2009) U-CARE: Utilities for performing goodness of fit tests and manipulating Capture-REcapture data. Ecography 32: 1071–1074 10.1111/j.1600-0587.2009.05968.x

[53] Péron G, Crochet P, Choquet R, Pradel R, Lebreton JD, et al (2010) Capture-recapture models with heterogeneity to study survival senescence in the wild. Oikos 119: 524–532 10.1111/j.1600-1706.2009.17882.x

[54] Pledger S, Pollock KH, Norris JL (2003) Open capture-recapture models with heterogeneity: I. Cormack-Jolly-Seber model. Biometrics 59: 786–794. 14969456 10.1111/j.0006-341x.2003.00092.x

[55] Pradel R, Choquet R, Lima MA, Merritt J, Crespin L (2010) Estimating population growth rate from capture–recapture data in presence of capture heterogeneity. J Agric Biol Environ Stat 15: 248–258 10.1007/s13253-009-0008-8

[56] Pradel R, Hines JE, Lebreton JD, Nichols JD (1997) Capture-recapture survival models taking account of transients. Biometrics 53: 60 10.2307/2533097

[57] Choquet R, Rouan L, Pradel R (2009) Program E-SURGE: a software application for fitting multievent models. In: Thomson DL, Cooch EG, Conroy MJ, editors. Modeling demographic processes in marked populations. New York: Springer. 845–865.

[58] Barrett-Lennard LG, Smith TG, Ellis GM (1996) A cetacean biopsy system using lightweight pneumatic darts, and its effect on the behavior of killer whales. Mar Mammal Sci 12: 14–27.

[59] Patenaude NJ, White BN (1995) Skin biopsy sampling of beluga whale carcasses: assessment of biopsy darting factors for minimal wounding and effective sample retrieval. Mar Mammal Sci 11: 163–171.

[60] Zerbini AN, Andriolo A, Heide-Jørgensen M-P, Pizzorno J, Maia Y, et al (2006) Satellite-monitored movements of humpback whales *Megaptera novaeangliae* in the Southwest Atlantic Ocean. Mar Ecol Prog Ser 313: 295–304 10.3354/meps313295

[61] Bailey H, Mate B, Palacios D, Irvine L, Bograd S, et al (2009) Behavioural estimation of blue whale movements in the Northeast Pacific from state-space model analysis of satellite tracks. Endanger Species Res 10: 93–106 10.3354/esr00239

[62] Hauser N, Zerbini AN, Geyer Y, Heide-Jørgensen M-P, Clapham P (2010) Movements of satellite-monitored humpback whales, *Megaptera novaeangliae*, from the Cook Islands. Mar Mammal Sci 26: 679–685 10.1111/j.1748-7692.2009.00363.x

[63] Reeb D, Best PB (2006) A biopsy system for deep-core sampling of the blubber of southern right whales, *Eubalaena australis* . Mar Mammal Sci 22: 206–213 10.1111/j.1748-7692.2006.00015.x

[64] Brown MR, Corkeron PJ, Hale PT, Schultz KW, Bryden MM (1994) Behavioral responses of east Australian humpback whales Megaptera novaeangliae to biopsy sampling. Mar Mammal Sci 10: 391–400 10.1111/j.1748-7692.1994.tb00496.x

[65] Gauthier J, Sears R (1999) Behavioral response of four species of balaenopterid whales to biopsy sampling. Mar Mammal Sci 15: 85–101 10.1111/j.1748-7692.1999.tb00783.x

[66] Kellar NM, Trego ML, Chivers SJ, Archer FI, Minich JJ, et al (2013) Are there biases in biopsy sampling? Potential drivers of sex ratio in projectile biopsy samples from two small delphinids. Mar Mammal Sci 29: E366–E389 10.1111/mms.12014

[67] Culik B, Adelung D, Woakes AJ (1990) The effect of disturbance on the heart rate and behavior of Adélie penguins (*Pygoscelis adeliae*) during the breeding season. In: Kerry KR, Hempel G, editors. Antarctic Ecosystems: Ecological Change and Conservation. Berlin: Springer-Verlag. 177–182.

[68] Wilson R, Culik B, Danfeld R, Adelung D (1991) People in Antarctica– how much do Adélie penguins *Pygoscelis adeliae* care? Polar Biol 11: 363–370 10.1007/BF00239688

[69] Regel J, Pütz K (1997) Effect of human disturbance on body temperature and energy expenditure in penguins. Polar Biol: 246–253.

[70] Wikelski M, Cooke SJ (2006) Conservation physiology. Trends Ecol Evol 21: 38–46 10.1016/j.tree.2005.10.018 16701468

[71] Tarlow EM, Blumstein DT (2007) Evaluating methods to quantify anthropogenic stressors on wild animals. Appl Anim Behav Sci 102: 429–451 10.1016/j.applanim.2006.05.040

[72] Winter Y, Stich KP (2005) Foraging in a complex naturalistic environment: capacity of spatial working memory in flower bats. J Exp Biol 208: 539–548 10.1242/jeb.01416 15671342

[73] Wolf M, Frair J, Merrill E, Turchin P (2009) The attraction of the known: the importance of spatial familiarity in habitat selection in wapiti *Cervus elaphus* . Ecography 32: 401–410 10.1111/j.1600-0587.2008.05626.x

[74] Ban SD, Boesch C, Janmaat KRL (2014) Taï chimpanzees anticipate revisiting high-valued fruit trees from further distances. Anim Cogn. doi:10.1007/s10071-014-0771-y. 10.1007/s10071-014-0771-y 24950721

[75] Marino L, Connor RC, Fordyce RE, Herman LM, Hof PR, et al (2007) Cetaceans have complex brains for complex cognition. PLoS Biol 5: e139 10.1371/journal.pbio.0050139 17503965 PMC1868071

[76] Gill J, Norris K, Sutherland W (2001) Why behavioural responses may not reflect the population consequences of human disturbance. Biol Conserv 97: 265–268.

[77] Frid A, Dill L (2002) Human-caused disturbance stimuli as a form of predation risk. Conserv Ecol 6: 11.

[78] Keith M, Bester MN, Bartlett PA, Baker D (2001) Killer whales (*Orcinus orca*) at Marion Island, Southern Ocean. African Zool 36: 163–175.

[79] Pistorius PA, Taylor FE, Louw C, Hanise B, Bester MN, et al (2002) Distribution, movement, and estimated population size of killer whales at Marion Island, December 2000. South African J Wildl Res 32: 86–92.

[80] Beale CM, Monaghan P (2004) Behavioural responses to human disturbance: a matter of choice? Anim Behav 68: 1065–1069 10.1016/j.anbehav.2004.07.002

[81] Williams R, Trites AW, Bain DE (2006) Behavioural responses of killer whales (*Orcinus orca*) to whale-watching boats: opportunistic observations and experimental approaches. J Zool 256: 255–270 10.1017/S0952836902000298

[82] Barbraud C, Weimerskirch H (2011) Assessing the effect of satellite transmitters on the demography of the wandering albatross *Diomedea exulans* . J Ornithol 153: 375–383 10.1007/s10336-011-0752-8

