# Supplementary figures and images for: Satellite Tagging and Biopsy Sampling of Killer Whales at Subantarctic Marion Island: Effectiveness, Immediate Reactions and Long-Term Responses
Source: PLoS One. 2014 Nov 6;9(11):e111835. doi: 10.1371/journal.pone.0111835 (PMC4222950; doi:10.1371/journal.pone.0111835)

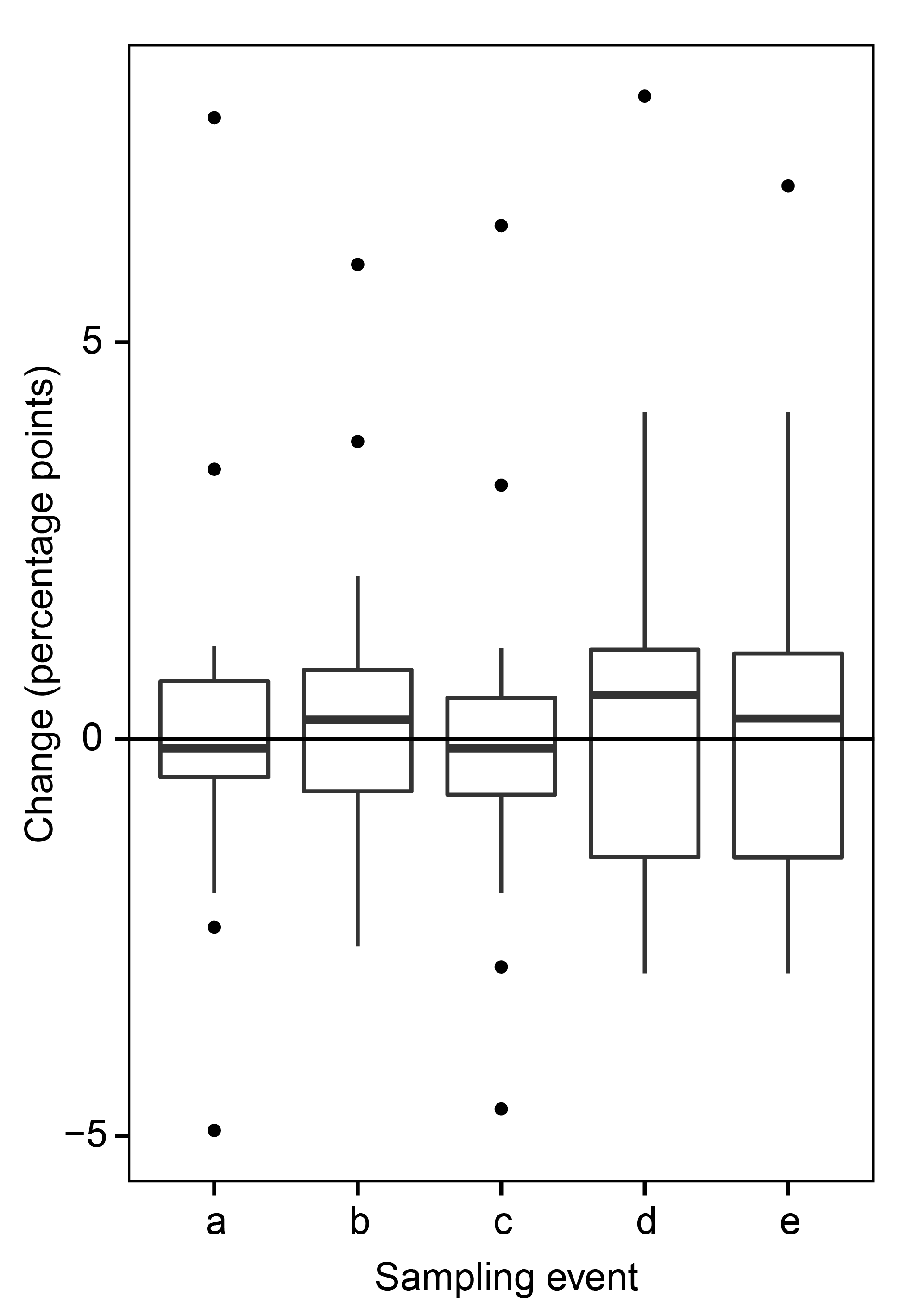

Supplement: Figure S1 — Changes (percentage points) in the sighting proportion of killer whales at Marion Island following various sampling events. a) tag or biopsy – first attempt; b) biopsy – first attempt; c) biopsy – first hit; d) tag – first attempt; e) tag – first hit. Sighting proportion (%) was calculated as the number of sightings of an individual during a given period, divided by the number of sightings of all individuals in the same period. Negative change thus indicates an individual was seen less following a sampling event. (TIF) [file pone.0111835.s001.tif]

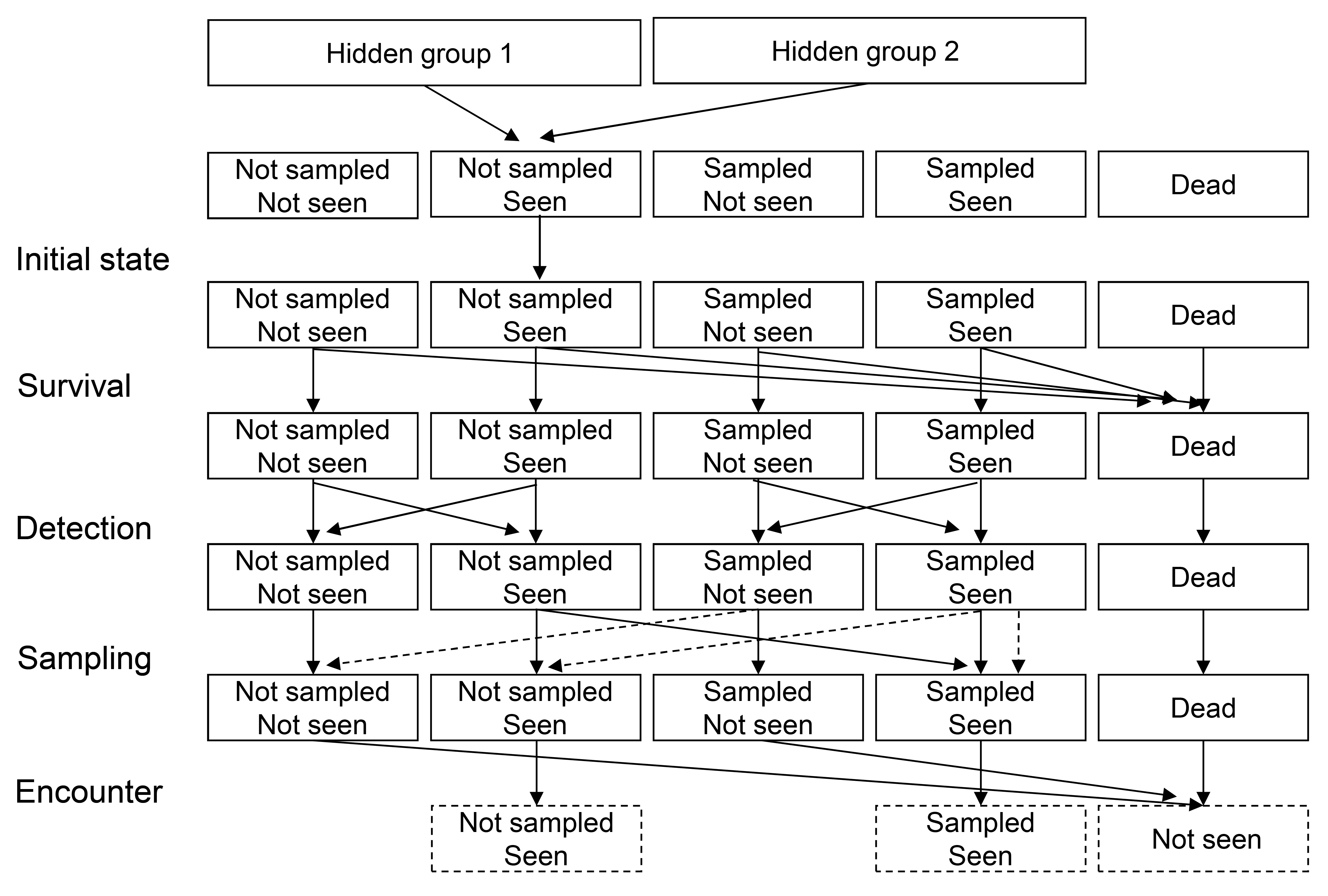

Supplement: Figure S2 — A multinomial tree diagram with arrows denoting the possible transitions between states (solid boxes) from t to t+1 . States occupied are not directly observed, but events (dashed boxes) represent observations following initial capture (‘Encounter’). Individuals belong to one of two hidden classes with distinct probabilities of detection; movement between detection groups over time is not allowed. Entry to the population conditions on the first encounter (‘Seen’) and all individuals are seen once or more prior to sampling (‘Initial state’ step). Subsequent state transition probabilities are decomposed in three steps as the product of the probabilities of ‘Survival’, ‘Detection’ and ‘Sampling’. Only individuals that are detected (‘Seen’) can be sampled. Once sampled, individuals either remain in the sampled state (permanent state change scenario; solid arrows) or may move back to the ‘Not Sampled’ state at the next occasion (mid-term sampling effect scenario; dashed arrows). (TIF) [file pone.0111835.s002.tif]
