# Supplementary material for: Satellite Tagging and Biopsy Sampling of Killer Whales at Subantarctic Marion Island: Effectiveness, Immediate Reactions and Long-Term Responses
Source: PLoS One. 2014 Nov 6;9(11):e111835. doi: 10.1371/journal.pone.0111835 (PMC4222950; doi:10.1371/journal.pone.0111835)
Supplement: Table S1 — Comparisons of sighting proportions before and after tagging and biopsy attempts on killer whales at Marion Island (paired Wilcox rank sum test). The sighting proportion is the number of photographic sightings of an individual in a given period, divided by the number of photographic sightings of all individuals in that period (following [1]). Notes: a N is the number of sampling attempts included for each comparison. b W is the test statistic. c Tag or biopsy – first attempt includes only the first attempt (regardless of whether it was a tag or biopsy attempt), hence it is not the sum of Tag – first attempt and Biopsy – first attempt. (DOCX) [file pone.0111835.s003.docx]

*Supplementary Table S1*

Comparisons of sighting proportions before and after tagging and biopsy attempts on killer whales at Marion Island (paired Wilcox rank sum test). The sighting proportion is the number of photographic sightings of an individual in a given period, divided by the number of photographic sightings of all individuals in that period (following [1]).

| Event | N^a^ | W^b^ | p-value |
| --- | --- | --- | --- |
| Tag or biopsy – first attempt^c^ | 30 | 506 | 0.4119 |
| Tag – first attempt | 16 | 134 | 0.8358 |
| Tag – first hit | 15 | 122 | 0.7089 |
| Biopsy – first attempt | 26 | 379 | 0.4618 |
| Biopsy – first hit | 24 | 329 | 0.4037 |

Notes: ^a^ *N* is the number of sampling attempts included for each comparison. ^b^ *W* is the test statistic. ^c^ *Tag or biopsy – first attempt* includes only the first attempt (regardless of whether it was a tag or biopsy attempt), hence it is not the sum of *Tag – first attempt* and *Biopsy – first attempt*.

1. Tezanos-Pinto G, Baker C (2012) Short-term reactions and long-term responses of bottlenose dolphins (*Tursiops truncatus*) to remote biopsy sampling. New Zeal J Mar Freshw Res 46: 13–29. doi:10.1080/00288330.2011.583256.
