# Supplementary material for: Satellite Tagging and Biopsy Sampling of Killer Whales at Subantarctic Marion Island: Effectiveness, Immediate Reactions and Long-Term Responses
Source: PLoS One. 2014 Nov 6;9(11):e111835. doi: 10.1371/journal.pone.0111835 (PMC4222950; doi:10.1371/journal.pone.0111835)
Supplement: Table S2 — Satellite tags deployed on killer whales at Marion Island showing the attachment duration, duty cycle and number of position estimates received. Notes: aSA – subadult, A – adult; b1– transmit 00∶00–24∶00 UTC, 2– transmit 00∶00–06∶00 and 12∶00–18∶00 UTC, 3– transmit 01∶00–22∶00 UTC for 30 days, thereafter 01∶00–22∶00 UTC on every second day, 4– transmit 01∶00–22∶00 UTC for 25 days, thereafter 01∶00–22∶00 UTC on every fourth day; cArgos position estimate quality class (see text for accuracy); d‘Accurate’ position estimates are quality class 1–3; number of accurate positions estimates per day was corrected for duty cycle (the proportion of time transmitting) and is thus expressed per ‘transmission day’, i.e., 24 transmission hours. (DOCX) [file pone.0111835.s004.docx]

*Supplementary Table S2*

Satellite tags deployed on killer whales at Marion Island showing the attachment duration, duty cycle number of position estimates received.

| Animal | | | PTT | Tag model | Duty cycle^b^ | Deployment date | Deployment length  (days) | Number of position estimates in each quality class^c^ | | | | | | | Accurate position estimates (.transmission day^-1^)^d^ |
| --- | --- | --- | --- | --- | --- | --- | --- | --- | --- | --- | --- | --- | --- | --- | --- |
| ID | Sex | Age class^a^ |  |  |  |  |  | A | B | 0 | 1 | 2 | 3 | Total |  |
| M053 | ? | SA | 67764 | Kiwisat 202 | 1 | 2011/06/04 | 0.8 | 4 | 5 | 2 | 3 | 2 | 2 | 18 | 8.7 |
| M007 | M | A | 107114 | Kiwisat 202 | 2 | 2011/11/05 | - | - | - | - | - | - | - | - | - |
| M030 | M | SA | 67845 | Kiwisat 202 | 2 | 2011/11/15 | 2.7 | 10 | 10 | 2 | 14 | 3 | 1 | 40 | 13.2 |
| M007 | M | A | 67768 | Kiwisat 202 | 1 | 2011/11/17 | 0.6 | 1 | 4 | 2 | 4 | 4 |  | 15 | 12.4 |
| M031 | F | A | 67847 | Kiwisat 202 | 2 | 2011/11/19 | 0.9 | 5 | 7 | 1 | 1 | 3 | 2 | 19 | 14.0 |
| M037 | F | A | 67848 | Kiwisat 202 | 2 | 2011/11/24 | 3.9 | 15 | 14 | 2 | 8 | 2 | 2 | 43 | 6.2 |
| M001 | M | SA | 67838 | Kiwisat 202 | 1 | 2011/12/02 | 1.8 | 11 | 9 | 3 | 9 | 6 | 2 | 40 | 9.7 |
| M007 | M | A | 115895 | SPOT5 | 3 | 2012/04/25 | 0.3 | 3 | 2 |  | 2 | 2 |  | 9 | 13.2 |
| M001 | M | SA | 115896 | SPOT5 | 3 | 2012/04/28 | 10.6 | 26 | 73 | 20 | 46 | 32 | 7 | 204 | 8.6 |
| M007 | M | A | 115897 | SPOT5 | 3 | 2012/05/01 | 53.2 | 167 | 214 | 122 | 305 | 297 | 78 | 1183 | 17.8 |
| M030 | M | SA | 115904 | SPOT5 | 3 | 2012/11/08 | 18.7 | 67 | 131 | 43 | 60 | 54 | 10 | 365 | 7.2 |
| M026 | F | A | 115903 | SPOT5 | 3 | 2012/11/29 | 29.7 | 89 | 224 | 33 | 110 | 83 | 30 | 569 | 8.2 |
| M059 | F | A | 115902 | SPOT5 | 3 | 2012/12/04 | 39.4 | 127 | 527 | 29 | 46 | 27 | 5 | 761 | 2.5 |
| M005 | M | A | 115901 | SPOT5 | 3 | 2012/12/14 | 16.0 | 78 | 131 | 37 | 71 | 61 | 17 | 395 | 10.1 |
| M035 | F | A | 115900 | SPOT5 | 3 | 2012/12/31 | 15.9 | 72 | 113 | 29 | 77 | 61 | 15 | 367 | 10.5 |
| M017 | F | A | 115899 | SPOT5 | 3 | 2013/01/12 | 40.0 | 177 | 483 | 75 | 106 | 76 | 26 | 943 | 6.5 |
| M001 | M | SA | 128704 | Mk10-A | 4 | 2013/04/23 | - | - | - | - | - | - | - | - | - |
| M049 | F | A | 128700 | Mk10-A | 4 | 2013/04/27 | 12.5 | 129 | 189 | 55 | 127 | 66 | 9 | 575 | 9.0 |
| M001 | M | SA | 128701 | Mk10-A | 4 | 2013/04/28 | 23.0 | 178 | 356 | 99 | 279 | 256 | 69 | 1237 | 14.6 |

Notes: ^a^ SA – subadult, A – adult; ^b^ 1 – transmit 00:00-24:00 UTC, 2 – transmit 00:00-06:00 and 12:00 – 18:00 UTC, 3 – transmit 01:00 – 22:00 UTC for 30 days, thereafter 01:00 – 22:00 UTC on every second day, 4 – transmit 01:00 – 22:00 UTC for 25 days, thereafter 01:00 – 22:00 UTC on every fourth day; ^c^ Argos position estimate quality class (see text for accuracy); ^d^ ‘Accurate’ position estimates are quality class 1-3; number of accurate positions estimates per day was corrected for duty cycle (the proportion of time transmitting) and is thus expressed per ‘transmission day’, i.e., 24 transmission hours.
