# Supplementary material for: Satellite Tagging and Biopsy Sampling of Killer Whales at Subantarctic Marion Island: Effectiveness, Immediate Reactions and Long-Term Responses
Source: PLoS One. 2014 Nov 6;9(11):e111835. doi: 10.1371/journal.pone.0111835 (PMC4222950; doi:10.1371/journal.pone.0111835)
Supplement: Table S3 — Approximate goodness of fit (GOF) tests for individual capture histories of killer whales at Marion Island. The overdispersion coefficient (ĉ) for a heterogeneity model including transience and trap-happiness was computed by removing the squared directional test statistics from the time dependant model [1]. (DOCX) [file pone.0111835.s005.docx]

*Supplementary Table S3*

Approximate goodness of fit (GOF) tests for individual capture histories of killer whales at Marion Island. The overdispersion coefficient (*ĉ*) for a heterogeneity model including transience and trap-happiness was computed by removing the squared directional test statistics from the time dependant model [1].

| Model | χ^2^ | df | *p*-level | ĉ |
| --- | --- | --- | --- | --- |
| Time dependant (CJS) | 221.48 | 92 | < 0.001 | 2.41 |
| Transient squared directional statistic | 2.50 | 1 | 0.012 | - |
| Trap-dependence squared directional statistic | -7.59 | 1 | < 0.001 | - |
| Heterogeneity of detection | 157.59 | 90 | < 0.001 | 1.75 |

1. Péron G, Crochet P, Choquet R, Pradel R, Lebreton JD, et al. (2010) Capture-recapture models with heterogeneity to study survival senescence in the wild. Oikos 119: 524–532. doi:10.1111/j.1600-1706.2009.17882.x.
