# Supplementary material for: Satellite Tagging and Biopsy Sampling of Killer Whales at Subantarctic Marion Island: Effectiveness, Immediate Reactions and Long-Term Responses
Source: PLoS One. 2014 Nov 6;9(11):e111835. doi: 10.1371/journal.pone.0111835 (PMC4222950; doi:10.1371/journal.pone.0111835)
Supplement: Table S4 — Multiple comparisons test (kruskalmc in R package pgirmess [1] ) results for significant reaction differences to tagging and biopsy attempts of various types. (DOCX) [file pone.0111835.s006.docx]

*Supplementary Table S4*

Multiple comparisons test (kruskalmc in R package pgirmess [1]) results for significant reaction differences to tagging and biopsy attempts of various types.

|  | | Biopsy | | Tag | |
| --- | --- | --- | --- | --- | --- |
|  |  | Hit | Miss | Hit | Miss |
| Biopsy | Hit | - |  |  |  |
|  | Miss | No | - |  |  |
| Tag | Hit | No | Yes | - |  |
|  | Miss | Yes | No | Yes | - |

1. Giraudoux P (2011) pgirmess: Data analysis in ecology. R package version 1.5.1. http://cran.r-project.org/web/packages/pgirmess/index.html
