# Supplementary material for: Satellite Tagging and Biopsy Sampling of Killer Whales at Subantarctic Marion Island: Effectiveness, Immediate Reactions and Long-Term Responses
Source: PLoS One. 2014 Nov 6;9(11):e111835. doi: 10.1371/journal.pone.0111835 (PMC4222950; doi:10.1371/journal.pone.0111835)
Supplement: Table S5 — Breeding populations of known killer whale prey at satellite tagging and biopsy sampling locations, and total breeding populations, at Marion Island. Seal numbers refer to pup production and penguin numbers to breeding pairs. Numbers in parentheses are percentage of the total breeding population. Dashes indicate zero animals. (DOCX) [file pone.0111835.s007.docx]

*Supplementary Table S5*

Breeding populations of known killer whale prey at satellite tagging and biopsy sampling locations, and total breeding populations, at Marion Island. Seal numbers refer to pup production and penguin numbers to breeding pairs. Numbers in parentheses are percentage of the total breeding population. Dashes indicate zero animals.

| Species | Rockhopper Bay | Transvaal Cove | Marion Island Total | Reference |
| --- | --- | --- | --- | --- |
| Southern elephant seal | 4 (0.7) | 14 (2.5) | 565 | Mammal Research Institute, unpublished data |
| Antarctic fur seal | - | - | 1 553 | Mammal Research Institute, unpublished data |
| Subantarctic fur seal | 105 (1.3) | 7 (0.1) | 8 323 | Mammal Research Institute, unpublished data |
| Macaroni penguin | 22 (0.0) | - | 356 136 | [1] |
| Rockhopper penguin | 200 (0.0) | - | 67 000 | [2] |
| King penguin | - | - | 165 000 | [3] |

1. Crawford RJM, Cooper J, Dyer BM (2003) Population of the macaroni penguin *Eudyptes chrysolophus* at Marion Island, 1994/95-2002/03, with information on breeding and diet. African J Mar Sci 25: 475–486. doi:10.2989/18142320309504036.
2. Crawford RJM, Cooper J, Dyer BM, Greyling MD, Klages NTW, et al. (2003) Decrease in numbers of the eastern rockhopper penguin *Eudyptes chrysocome filholi* at Marion Island, 1994/95-2002/03. African J Mar Sci 25: 487–498. doi:10.2989/18142320309504037.
3. Crawford RJM, Cooper J, Dyer BM, Greyling MD, Klages NTW, et al. (2003) Populations of surface-nesting seabirds at Marion Island, 1994/95-2002/03. African J Mar Sci 25: 427–440. doi:10.2989/18142320309504032.
