# Supplementary material for: Satellite Tagging and Biopsy Sampling of Killer Whales at Subantarctic Marion Island: Effectiveness, Immediate Reactions and Long-Term Responses
Source: PLoS One. 2014 Nov 6;9(11):e111835. doi: 10.1371/journal.pone.0111835 (PMC4222950; doi:10.1371/journal.pone.0111835)
Supplement: Methods S1 — Further information about field methods used. (DOCX) [file pone.0111835.s011.docx]

**SUPPLEMENTARY METHODS S1**

**Field methods**

We used a 68 kg draw weight recurve crossbow (Barnett Panzer V; Barnett Outdoors, LLC, Tarpon Springs, Florida, United States of America) equipped with a red dot sight (Gamo 30 mm; Gamo Outdoor, S.L., Barcelona, Spain) for biopsy sampling and satellite tagging. Carbon fibre bolts/arrows (length = 508 - 559 mm; Barnett Headhunter and TenPoint Pro Elite; TenPoint Crossbow Technologies, Mogadore, Ohio, United States of America) were tethered with monofilament or braided line (22 - 36 kg break strength, Double X Hi-Abrasion and Daiwa Saltiga Boat Braid) and a fishing reel (Shimano Alivio 4000 RC; Shimano Inc., Sakai City, Osaka, Japan and Daiwa Silvercast 100; Daiwa Corp., Cypress, California, United States of America) mounted on the crossbow [1]. The bolts were fitted with conical or cylindrical high density foam floats (length = 65 mm, maximum diameter = 30 mm).

We obtained tissue samples using biopsy tips/cutting heads attached to the bolts (described above) with threaded connectors. The biopsy tips are stainless steel cylinders (length = 25 mm, outside diameter = 9 mm, inside diameter = 7 mm) with a sharp bevelled cutting edge and three short (3 mm), rearward facing barbs to retain the tissue sample. A hole in the side of the biopsy tip prevents pressure buildup as the dart strikes the skin, and a stainless steel flange prevents penetration beyond 25 mm [1].

Biopsy tips were cleaned and sterilized by first rinsing in fresh water and removing macroparticles with a brush and then either boiling them in distilled water for 20 minutes and flame sterilizing them twice with 100% ethanol or by placing them in 5% sodium hypochlorite for 10 minutes, followed by 10 minutes in 100% ethanol and then 10 minutes in distilled water. Sterile tips were placed in sealed plastic bags until use.

The tissue samples obtained were processed within 30 minutes of biopsy sampling. Biopsy samples were sectioned into three subsamples: the first subsample was stored in 20% DMSO saturated with NaCl [2] at -20° C for genetic analysis [3, 4] and the other two subsamples were stored without preservative at -80° C for fatty acid and stable isotope analyses.

**References**

1. Lambertsen RH (1987) A biopsy system for large whales and its use for cytogenetics. J Mammal 68: 443–445. doi:10.2307/1381495
2. Amos W, Hoelzel AR (1991) Long-term preservation of whale skin for DNA analysis. Rep Int Whal Comm special issue 13: 99–104.
3. Moura AE, Kenny JG, Chaudhuri R, Hughes M, Reisinger RR, de Bruyn PJN, Dahlheim ME, Hall N, Hoelzel AR (2014) Phylogenomics of the killer whale indicates ecotype divergence in sympatry. Heredity. doi:10.1038/hdy.2014.67
4. Moura AE, Kenny JG, Chaudhuri R, Hughes MA, Welch A, Reisinger RR, de Bruyn PJN, Dahlheim ME, Hall N, Hoelzel AR (2014) Population genomics of the killer whale indicates ecotype evolution in sympatry involving both selection and drift. Mol Ecol. doi:10.1111/mec.12929
